# Does Sensory Retraining Improve Sensation and Sensorimotor Function Following Stroke: A Systematic Review and Meta-Analysis

**DOI:** 10.3389/fnins.2019.00402

**Published:** 2019-04-30

**Authors:** Ines Serrada, Brenton Hordacre, Susan L. Hillier

**Affiliations:** ^1^School of Health Sciences, University of South Australia, Adelaide, SA, Australia; ^2^Division of Health Sciences, University of South Australia, Adelaide, SA, Australia

**Keywords:** stroke, rehabilitation, sensory, physiotherapy, occupational therapy, recovery of function

## Abstract

**Background:** Reduced sensation is experienced by one in two individuals following stroke, impacting both the ability to function independently and overall quality of life. Repetitive activation of sensory input using active and passive sensory-based interventions have been shown to enhance adaptive motor cortical plasticity, indicating a potential mechanism which may mediate recovery. However, rehabilitation specifically focusing on somatosensory function receives little attention.

**Objectives:** To investigate sensory-based interventions reported in the literature and determine the effectiveness to improve sensation and sensorimotor function of individuals following stroke.

**Methods:** Electronic databases and trial registries were searched from inception until November 2018, in addition to hand searching systematic reviews. Study selection included randomized controlled trials for adults of any stroke type with an upper and/or lower limb sensorimotor impairment. Participants all received a sensory-based intervention designed to improve activity levels or impairment, which could be compared with usual care, sham, or another intervention. The primary outcomes were change in activity levels related to sensorimotor function. Secondary outcomes were measures of impairment, participation or quality of life.

**Results:** A total of 38 study trials were included (*n* = 1,093 participants); 29 explored passive sensory training (somatosensory; peripheral nerve; afferent; thermal; sensory amplitude electrical stimulation), 6 active (sensory discrimination; perceptual learning; sensory retraining) and 3 hybrid (haptic-based augmented reality; sensory-based feedback devices). Meta-analyses (13 comparisons; 385 participants) demonstrated a moderate effect in favor of passive sensory training on improving a range of upper and lower limb activity measures following stroke. Narrative syntheses were completed for studies unable to be pooled due to heterogeneity of measures or insufficient data, evidence for active sensory training is limited however does show promise in improving sensorimotor function following stroke.

**Conclusions:** Findings from the meta-analyses and single studies highlight some support for the effectiveness of passive sensory training in relation to sensory impairment and motor function. However, evidence for active sensory training continues to be limited. Further high-quality research with rigorous methods (adequately powered with consistent outcome measures) is required to determine the effectiveness of sensory retraining in stroke rehabilitation, particularly for active sensory training.

## Introduction

### Rationale

Sensation is the means by which we process and interact with the world and our environment (Connell, 2007; Carey et al., 2016). It allows us to detect and discriminate objects and textures, know where our body is in space (proprioception) and accurately perceive and discriminate sensations of pain, temperature, pressure and vibration (Carey, 1995; Schabrun and Hillier, 2009; Doyle et al., 2010; Carey et al., 2011, 2018). As a result, sensation is critical for normal human function and is fundamental for motor behaviors (Doyle et al., 2010). For example, somatosensory input is required for accurate and adaptable motor control and the acquisition of motor skills, suggesting intact sensation may be a critical component to facilitate motor rehabilitation (Carey et al., 1993; Yekutiel and Guttman, 1993; Wu et al., 2006; Celnik et al., 2007).

Reduced sensation is experienced by one in two individuals following stroke (Carey et al., 2018), impacting both the ability to function independently and overall quality of life (Carey et al., 1993, 2018; Yekutiel and Guttman, 1993). Most significantly these deficits contribute to confidence and movement difficulties with an enduring impact on simple everyday activities such as reaching, grasping and manipulating objects or knowing where a foot is positioned during gait without the need to visually observe its position. As expected, reduced sensation following stroke is associated with slower recovery, reduced motor function (in terms of quality of movement control) and lesser rehabilitation outcomes (Wu et al., 2006; Doyle et al., 2010; de Diego et al., 2013; Carey, 2014). These deficits continue to persist for years with many individuals often learning not to use their sensory affected limb (learned non-use) due to uncertainty, lack of confidence of whether to use it and/or vulnerability and fear of safety (Doyle et al., 2010; Turville et al., 2017). This continued disuse leads to a further reduction and deterioration (Carey et al., 1993, 2018; Yekutiel and Guttman, 1993; Doyle et al., 2010). In addition, these sensory deficits have widespread effects not only in predicting poor functional outcomes but increasing length of hospitalization, reduced numbers of discharges to home and increased mortality rates (Yekutiel and Guttman, 1993; Carey, 1995; Doyle et al., 2010; Carey et al., 2011).

Repetitive activation of sensory input (sensory-based interventions) has been shown to enhance adaptive motor cortical plasticity, indicating a potential mechanism which may mediate recovery (Carrico et al., 2016b). As such, sensory input may be integral to facilitate the recovery of function following stroke (Schabrun and Hillier, 2009). Yet despite these findings suggesting an association between sensory and motor function in recovery following stroke, rehabilitation specifically focusing on somatosensory function receives little attention (Carey, 1995; Schabrun and Hillier, 2009; de Diego et al., 2013; Carey et al., 2016).

### Objectives/Research Question

The objective of this study was to systematically review and update the literature around somatic sensory-based interventions to improve sensation and sensorimotor function of individuals following stroke. This review is an extension of (Schabrun and Hillier, 2009).

## Methods

### Systematic Review Protocol

The protocol was specified a priori and according to the Preferred Reporting Items for Systematic Reviews and Meta-Analyses Protocols. This study was registered prospectively on November 23, 2018, with the International Prospective Register of Systematic Reviews before commencement (CRD42017078103); http://www.crd.york.ac.uk/PROSPERO/display_record.php?ID=CRD42017078103.

### Study Design and Eligibility Criteria

Database searching was conducted based on the predetermined criteria in Table 1.

**Table 1:** Search criteria.

**Variable**	**Criteria**
Population	Adults (>18 years) following a stroke with a sensory and/or motor deficit. Any type (ischemic/ hemorrhage), location and stage (acute, sub-acute, chronic) of stroke.
Intervention	**Inclusion/** Sensations of interest were limited to somatic (cutaneous, and proprioceptive). Any sensory training (active, passive, hybrid) applied to the upper/lower limb or trunk, delivered as stand-alone or an adjunct to usual care and addressing the recovery of sensation and/or sensorimotor function. *Passive: An externally applied sensory stimulation approach, with a purported mechanism of priming the nervous system. Sensory stimulation to produce activation of cutaneous nerves in the absence of muscle contraction (sub-motor) with a clear intent to stimulate only somatosensory afferents (e.g., thermal stimulation, pressure, peripheral nerve stimulation, transcutaneous electrical nerve stimulation, vibration stimulation). Active: A sensory retraining approach based on graded re-education using learning principles and augmenting sensory input (e.g., proprioception, tactile recognition, desensitization, stereognosis, localization, discrimination). Hybrid: A combination of sensory stimulation (passive) and retraining (active) interventions (e.g., haptic-based augmented reality, feedback devices that augment targeted sensory afferents)*. **Exclusion/** Studies which combine sensory training with other forms of therapy or where sensory training is embedded within broader rehabilitation protocols – in either case the effects of the sensory program cannot be isolated from the potential effects of the other approaches. *Passive: Functional/neuromuscular electrical stimulation (targets motor efferents), paired associative, acupuncture or dermatomal stimulation, brain stimulation (transcranial magnetic/peripheral magnetic or transcranial direct current stimulation). Active: Mirror therapy, brain computer interface, visual-based robotics/virtual-augmented reality, biofeedback (forceplates), kinematics/whole body vibration, manipulating/varying multi-modal sensation (balance training that includes manipulating vision)*.
Comparator	Any inactive (placebo/sham, no treatment) or active control (usual care).
Outcome	Primary outcome: Change in activity levels related to sensorimotor function (measures of mobility, upper/lower limb function and task-specific activities). Secondary outcomes: Measures of motor impairment (range of motion, strength or postural sway), participation and quality of life. Change in sensory impairment as measured by a standardized sensory test (Nottingham Sensory Assessment).
Design	Randomized Controlled Trials.
Publication/Date Language	No limits applied. No limits applied. Studies in languages other than English were translated.

Within sensory training the types of interventions and the mechanism of action differ significantly making it difficult to clearly delineate intervention effects. Sensations of interest were limited to somatic (cutaneous and proprioceptive). Sensory training was separated into three areas; passive (an externally applied sensory stimulation approach, with a purported mechanism of priming the nervous system), active (a sensory retraining approach based on graded re-education using learning principles) and hybrid (a combination of sensory stimulation and retraining) interventions (see Table 1) (Schabrun and Hillier, 2009; Doyle et al., 2010).

### Search Strategy and Data Sources

The search strategy of medical subject headings and keywords were developed in Ovid Medline database using variations of the term stroke and sensation, “sensory training,” “sensory education,” “sensory rehabilitation,” “sensory practice,” “sensory treatment,” “sensory awareness,” “sensory movement,” “sensory intervention,” “sensory discrimination,” “stimulation therapy,” “cutaneous stimulation,” “electrical stimulation,” “afferent stimulation,” “sensory stimulation,” “stimulation therapy,” “somatosensory stimulation” (see Appendix 1 in Supplementary Material). An academic librarian with experience in health-related systematic reviewing was also consulted. This strategy was adapted for other bibliographic databases, database-specific filters were applied and modifications were restricted to closely reflect the original strategy. Eleven electronic databases were searched from inception to November 27, 2018: AMED, CINAHL, Cochrane Database of Systematic Reviews, Elsevier Scopus, Embase, Medline, OTseeker, Ovid Emcare, PEDRO and Pubmed. Five trial registries were searched with studies documented and followed for published results: Australian New Zealand Clinical Trials Registry, ClinicalTrials.gov, Cochrane Central Register of Controlled Trials, Stroke Trials Registry and World Health Organization International Clinical Trials Registry Platform. A citation-tracking database of Web of Science was used as well as hand searching reference lists of included studies, systematic reviews, clinical guidelines and key reviews in this area to identify individual trials not retrieved from the electronic search. To complete word citation tracking, key references were entered in Science Citation.

### Study Selection

Search result records were saved in EndNote X8 and Covidence online software. Duplicate publications were identified and removed. Studies retrieved were screened and assessed by one reviewer for the obviously irrelevant titles. Studies were then assessed for meeting the selection criteria based on title and abstract. Of the eligible studies, full texts were accessed and independently assessed by two reviewers (I.S. and B.H.). Disagreement between reviewers was discussed to reach consensus and/or resolved by a third reviewer (S.H.).

### Data Extraction

Data extraction was conducted using the *Cochrane Handbook* version 5.1.0 recommendations, using a predesigned data extraction spreadsheet in Microsoft Excel 2018, version 16.16.5. Extracted data included characteristics of participants, intervention, comparator, and outcome results.

### Risk of Bias Analysis

Two reviewers (I.S. and B.H.) independently assessed all the included studies using the standardized domain-based evaluation Cochrane Risk of Bias Tool, the preferred tool of the Cochrane Collaboration (Higgins et al., 2011). Assessments were completed using Covidence online software to blind judgements of reviewers. Disagreement between reviewers was discussed to reach consensus and resolved by a third reviewer (S.H.).

### Data Synthesis and Statistical Analysis

Descriptive statistics were used to summarize findings of the included studies. Data including study characteristics/method (study design, participants, intervention, controls and outcome measures) and results (sample size, means and standard deviations) where appropriate were manually extracted by two independent assessors (I.S. and S.H.) and transferred into Microsoft Excel 2018 (version 16.16.5). Review Manager (RevMan 5.3.5) software was used for data synthesis and to perform meta-analyses with sufficiently homogenous data to calculate effect sizes. In the meta-analyses, data from randomized controlled trials were pooled based on comparable control groups and outcome measures. These were then grouped into the *International Classification of Functioning, Disability and Health* framework outcomes with the primary interest of activity levels (for example Wolf Motor Function Test or Berg Balance Scale) and secondarily impairment (for example range of motion or strength). Two authors (I.S. and S.H.) extracted and entered data and cross-referenced to reduce risk of errors. The mean and standard deviation data from the first post intervention time-point (or first group from crossover studies) were used. Data from time-points other than the first post intervention assessment including follow-up data were not analyzed because of the heterogeneity between studies. When mean and standard deviation data were not available, study authors were contacted for the original data set. Those that could not be contacted but median and interquartile range were available, a formula for the standard deviation (SD) from Hillier and Inglis-Jassiem *SD* = 0.75 × IQR (*SD* = 0.75 × IQR) was used and it was assumed the median equated to the mean (Liang et al., 2012). If appropriate data was still not possible, the study was excluded from meta-analyses. Either post-intervention means or mean change scores were included. In the case of dichotomous data, number of participants in both the experimental and control group and the total sample size were identified. The data were generally ordinal and analyzed as continuous data outcomes using the summary statistics recommended by the Cochrane Collaboration. Data were then analyzed to calculate either relative risk, with 95% confidence intervals or individual and group effect sizes. Meta-analyses used the fixed-effect model, analysis of effect sizes used the mean difference (MD). Heterogeneity was assessed with the *I*^2^ test, where >50% was interpreted as substantial heterogeneity. Where data were not available or of unacceptable heterogeneity, a narrative summary of study results was produced using reported effects.

## Results

### Study Selection and Characteristics

**Figure 1:**
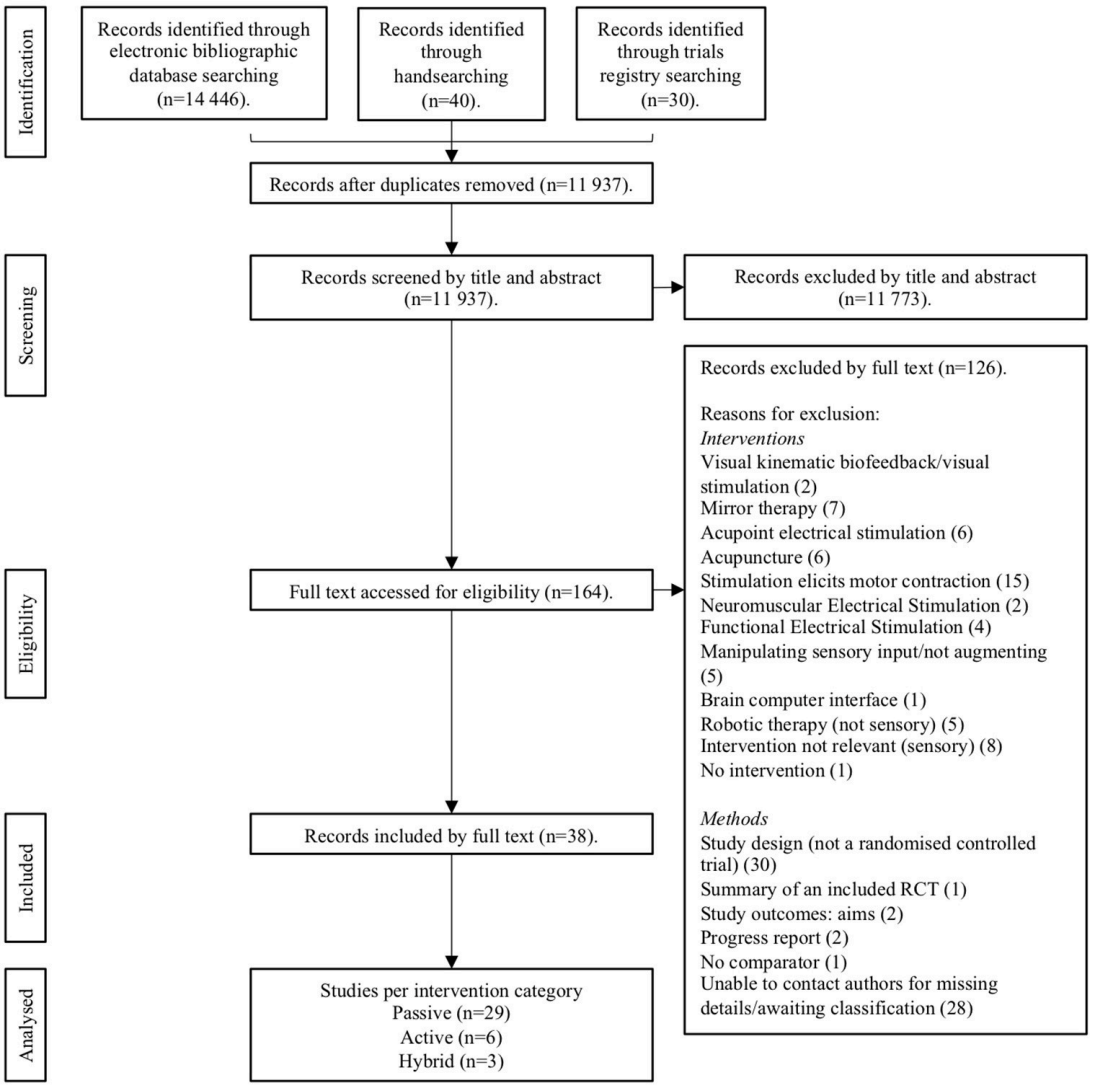
Preferred Reporting for Systematic Reviews and Meta-Analyses (PRISMA) flow diagram of the review process.

A total of 14,446 trials were identified from preliminary searches, with the summary flow of trials outlined in Figure 1. The final analysis included 38 papers, of these 29 passive (20 upper limb and 9 lower limb); 6 active (4 upper limb and 2 lower limb) and 3 hybrid studies (2 upper limb and 1 trunk) (see **Tables 4**–**6**). A total sample of 1,093 participants were included. Total mean age range was 39.9–72.6 years, 657 of these were males and 399 females and 366 were affected on the left-side and 404 right. Total mean time since stroke ranged from 0.87 months to 11.5 years. The most common reasons for exclusion are reported in Figure 1.

### Synthesized Findings

#### Meta-Analyses

Where possible, data were pooled based on outcome measures and controls. Meta-analysis of data to determine effectiveness was possible for 13 studies (11 passive, 2 active) (11 upper limb, 2 lower limb) (see Tables 2, 3) with a total sample of 385 participants included. Pooling was not possible for the remaining 25 studies because of the diversity of intervention protocols and outcome measures, the results of these interventions are reported narratively.

**Table 2:** Effect size (95% CIs) for sensory training compared to usual care.

**Study (author, year)**	**Outcome measure**	**Total sample size E:C (n)**	**Mean difference (IV, Fixed, 95% CI)**	**Heterogeneity (*I*^**2**^)**	**Overall effect (P)**
**ACTIVITY**					
Chen et al., 2011; Liang et al., 2012	Functional Ambulation Category	32:31	0.71 (0.59, 0.82)	0%	*P* < 0.00001
Chen et al., 2011; Liang et al., 2012	Motor Assessment Scale	32:31	6.15 (4.91, 7.40)	0%	*P* < 0.00001
Chanubol et al., 2012; Lin et al., 2014a,b	Box and Block Test	42:42	2.28 (-4.62,9.17)	0%	*p* = 0.52
Chanubol et al., 2012; Liang et al., 2012	Barthel Index	35:35	8.27 (5.59, 10.95)	95%	*p* < 0.00001
Chen et al., 2011; Liang et al., 2012	Berg Balance Scale	32:31	−3.78 (-6.39, −1.18);	94%	*p* = 0.004
**IMPAIRMENT**					
Chen et al., 2011; Liang et al., 2012; de Diego et al., 2013	Fugl-Meyer Assessment	44:40	5.93 (5.17, 6.70)	91%	*P* < 0.00001

**Table 3:** Effect size (95% CIs) for sensory training compared to sham stimulation.

**Study (author, year)**	**Outcome measure**	**Total sample size E:C (n)**	**Mean difference (IV, Fixed, 95% CI)**	**Heterogeneity (*I*^**2**^)**	**Overall effect (P)**
**ACTIVITY**					
Stein et al., 2010; Wu et al., 2010; Fleming et al., 2015; Carrico et al., 2016a,b	Action Research Arm Test	71:70	2.80 (2.27, 3.32)	62%	*p* < 0.00001
Carrico et al., 2016a,b	Wolf Motor Function Test	28:27	−0.13 (−0.22, 0.04)	0%	*p* = 0.006
Stein et al., 2010; Sullivan et al., 2012; Fleming et al., 2015	Motor Activity Log	51:50	0.01 (−0.32, 0.34)	0%	*P* = 0.96
Stein et al., 2010; Sullivan et al., 2012	Stroke Impact Scale	35:33	−1.86 (−5.85, 2.12)	0%	*p* = 0.36
**IMPAIRMENT**					
Cambier et al., 2003; Stein et al., 2010; Sullivan et al., 2012; Fleming et al., 2015; Carrico et al., 2016a,b	Fugl-Meyer Assessment	90:89	2.75 (1.53, 3.96)	35%	*p* < 0.00001
Cambier et al., 2003; Sullivan et al., 2012	Nottingham Sensory Assessment	31:30	0.59 (0.75, 1.93)	84%	*p* = 0.39

##### Sensory versus usual care

Of the data pooled based on comparisons of sensory versus usual care (see Table 2), a significant difference favoring sensory training was found on the Functional Ambulation Category (FAC) from two studies investigating passive lower limb sensory interventions (thermal stimulation) (Chen et al., 2011; Liang et al., 2012) (MD, fixed effects 0.71; 95% CI 0.59, 0.82; *z* = 12.35; *P* = 0.00001). A significantly positive difference was also found for the Motor Assessment Scale (MAS) (MD, fixed effects 6.15; 95% CI 4.91, 7.40; *z* = 9.69; *P* = 0.00001). The Box and Block Test (BBT) did not show a significant effect (MD, fixed effects 2.28; 95% CI −4.62,9.17; *z* = 0.65; *p* = 0.52) with one active upper limb study (Perfetti's method) (Chanubol et al., 2012) showing a slight positive effect while the two passive upper limb studies (somatosensory and afferent stimulation) (Lin et al., 2014a,b) showed no effect (see Appendix 2 in Supplementary Material for figures). Comparisons of these outcomes FAC (*I*^2^ = 0%), MAS (*I*^2^ = 0%) and BBT (*I*^2^ = 0%) showed no heterogeneity.

The Barthel Index (BI) showed an overall positive significant effect from sensory training (MD, fixed effects 8.27; 95% CI 5.59, 10.95; *z* = 6.05, *p* = 0.00001), the passive lower limb study (thermal stimulation) (Liang et al., 2012) favored sensory training while the active upper limb study (Perfetti's method) was equivocal (Chanubol et al., 2012). The Berg Balance Scale (BBS) meta-analysis showed a significantly negative result, with both passive lower limb studies (thermal stimulation) favoring the control group (Chen et al., 2011; Liang et al., 2012) (MD, fixed effects, −3.78; 95% CI −6.39, −1.18; *z* = 2.84; *p* = 0.004). A significant effect favoring sensory training was found on the Fugl-Meyer Assessment (FMA) (MD, fixed effects 5.93; 95% CI 5.17, 6.70; *z* = 15.21; *P* = 0.00001) with two lower limb passive studies (thermal stimulation) (Chen et al., 2011; Liang et al., 2012) indicating a positive change while the third, an active upper limb study (de Diego et al., 2013) reported a negative effect (see Appendix 2 in Supplementary Material for figures). Comparisons of these outcomes BI (*I*^2^ = 95%), BBS (*I*^2^ = 94%), and FMA (*I*^2^ = 91%) showed considerable heterogeneity.

##### Sensory versus sham stimulation

Further meta-analyses were conducted for the comparison of sensory versus sham stimulation (see Table 3). For the outcomes of Action Research Arm Test (ARAT) (MD, fixed effects 2.80; 95% CI 2.27, 3.32; *z* = 10.46, *p* = 0.00001), Wolf Motor Function Test (WMFT) (MD, fixed effects −0.13; 95% CI −0.22, 0.04; *z* = 2.73, *p* = 0.006) and FMA (MD, fixed effects 2.75; 95% CI 1.53, 3.96; *z* = 4.43, *p* = 0.00001) all returned a significant effect (see Appendix 3 in Supplementary Material for figures). Heterogeneity was variable with substantial heterogeneity reported for ARAT (*I*^2^ = 62%), moderate for FMA (*I*^2^ = 35%) and no heterogeneity for WMFT (*I*^2^ = 0%).The MAL (MD, fixed effects 0.01; 95% CI −0.32, 0.34; *z* = 0.05; *P* = 0.96) and Nottingham Sensory Assessment (NSA) (MD, fixed effects 0.59; 95% CI −0.75, 1.93; *z* = 0.86, *p* = 0.39) showed no significant effect on sensory training, while Stroke Impact Scale (SIS) (MD, fixed effects −1.86 (95% CI −5.85, 2.12) *z* = 0.92, *p* = 0.36) returned a negative effect (see Appendix 3 in Supplementary Material for figures). No heterogeneity was reported for MAL (*I*^2^ = 0%) and SIS (*I*^2^ = 0%), while considerable heterogeneity was indicated for NSA (*I*^2^ = 84%).

#### Narrative Synthesis

Narrative synthesis was used to summarize the randomized controlled trials that could not be pooled in meta-analyses.

##### Passive sensory training interventions

Passive sensory training interventions used a variety of frequency parameters and intensities (see Table 4). Five studies applied transcutaneous electrical nerve stimulation (TENS), the three lower limb studies indicated positive results for balance and mobility (Tyson et al., 2013; Ng et al., 2016; In and Cho, 2017), while the two upper limb studies showed no change for hemineglect (Polanowska et al., 2009; Seniow et al., 2016). Two studies used sensory amplitude electrical stimulation (SES), both studies showed a slight positive effect with upper limb motor function and sensation (Sullivan et al., 2012) and lower limb spasticity and gait (Yavuzer et al., 2007) however these were not significant. Three upper limb studies applied repetitive peripheral nerve stimulation (RPSS), two of these studies showed positive findings on hand function (dos Santos-Fontes et al., 2013), however for Conforto 2010 this was observed only in the lower intensity group (Conforto et al., 2010). While contradictory results were found for pinch strength (Klaiput and Kitisomprayoonkul, 2009; Conforto et al., 2010) and no effect on arm function (Klaiput and Kitisomprayoonkul, 2009). Five upper limb studies used peripheral nerve stimulation (PNS), two studies showed a slight positive effect on hand function (Wu et al., 2006; Celnik et al., 2007). While three studies showed positive findings on arm function (Ikuno et al., 2012; Carrico et al., 2016a,b). Five studies used thermal stimulation, the three upper limb studies showed positive findings on arm function (Wu et al., 2010) and motor function, spasticity, range and sensation (Chen et al., 2005, 2011). Similarly, the two lower limb studies highlighted positive effects on motor function and spasticity (Liang et al., 2012; Hsu et al., 2013). Of the two median nerve stimulation (MNS) studies, both studies indicated positive effects on hand function and pinch force (Conforto et al., 2002, 2007). Two studies using somatosensory stimulation (SS) showed positive improvements on arm and hand function (Lin et al., 2014b; Fleming et al., 2015), while the third study using afferent stimulation in addition also improved in gait and mobility (Lin et al., 2014a). A single upper limb study combined subsensory electrical and vibratory stimulation, no significant effect on arm function was found (Stein et al., 2010). While another upper limb study used a splint connected to an intermittent pneumatic compression device showed positive results on sensation, motor function and spasticity (Cambier et al., 2003). Of two lower limb studies, one provided a vibration stimulus showing improvements in postural sway and gait ability (Lee et al., 2013), while the other study delivered low-amplitude segmental muscle stimulation with positive results on mobility and gait parameters (Paoloni et al., 2010).

**Table 4:** Passive sensory training study characteristics.

**Author, year**	**Study design**	**Sample size**	**Age (years) mean (SD)**	**Gender (M:F)**	**Stroke duration (time since stroke) mean (SD)**	**Side of stroke (affected side, L: R)**	**Frequency, pulse length, intensity**	**Target duration**	**Control condition**	**Outcome measures**	**Direction of effects (+ positive, – negative, +/− both)**
Cambier et al., 2003	RCT (Parallel Group)	11E, 12C	63.9 ± 11.2E, 61.1 ± 12.8C	5:6E, 9:3C	114.1 ± 92.6E, 83.2 ± 44.9C (days)	6:5E, 8:4C	Pneumatic compression (10 cycles of 3 min with a peak of 40 mmHg)	UL 30 min, 5 days/week for 4 weeks, 20 sessions	Sham SWT and standard PT	NSA, FMA-UE, AS, VAS	+
Carrico et al., 2016a	RCT (Parallel Group)	10E, 9C	56.7E, 54.6C	3:7E, 6:3C	29.5 (months)	6:4E, 2:7C	PNS 10 Hz, 1 ms Mild paraesthesias	UL (posterior interosseous, median and ulnar nerves) 10 × 2 h consecutive sessions	Sham PNS paired with/preceding modified constraint induced movement therapy	WMFT, FMA-UE, ARAT	+
Carrico et al., 2016b	RCT (Parallel Group)	18E, 18C	58.7 ± 12.1E, 65.4 ± 10.8C (months)	9:9E, 9:9C	39.2 ± 34.6E, 25.7 ± 17.7C (months)	6:12E, 3:15C	PNS 10 Hz, 1 ms Mild paraesthesias	UL (radial and median nerves) 10 × 2h consecutive sessions	Sham PNS paired with/preceding intensive task oriented training	FMA-UE, WMFT, ARAT	+
Celnik et al., 2007	RCT (Crossover)	9	55.2 ± 14.3	3:6	3.2 ± 1.6 (years)	–	PNS 10 Hz, 1 ms Mild paraesthesias	UL (hand) 1 × 2 h session	No stimulation	JTHFT	+
Chen et al., 2005	RCT (Parallel Group)	15E, 14C	58.5 ± 12.9E, 59.6 ± 12.0C	6:9E, 10:4C	14.3 ± 6.8E, 12.4 ± 6.6 C (days)	10:5E, 8:6C	Thermal stimulation (10 × [30s + 30 s] × 2 × 2)	UL (wrist and hand) 20–30 min, 5 days/week for 6 weeks, 30 sessions	Standard rehabilitation	Brunnstrom stages, MMAS, MAS, monofilaments, HGS, wrist E/F angles	+/−
Chen et al., 2011	RCT (Parallel Group)	17E, 16C	58.0 ± 11.5E, 62.3 ± 11.3C	13:4E, 9:7C	11 (7.13-9.0)E, 11 (6.98–10.5)C (days)	11:6E, 9:7C	Thermal stimulation (8 × [30 s + 30 s] × 2 × 3)	LL (calf or foot) 30–40 min, 5 days/week for 6 weeks, 30 sessions	Standard rehabilitation	FMA-LE, MRC LE scale, MMAS, PASS, BBS, FAC, MAS	+
Conforto et al., 2002	RCT (Crossover)	8	65 (38-81)	7:1	66 (14–84) (months)	n/a	MNS 10 Hz, 1 ms Strong paresthesias	UL (median nerve) 1 × 2 h session	No stimulation	Pinch strength	+
Conforto et al., 2007	RCT (Crossover)	11	39.9 (4.2)	4:7	4.3 (0.7) (years)	9:2	MNS 10 Hz, 1 ms Strong paraesthesias	UL (median nerve) 1 × 2 h session	Subthreshold low-frequency stimulation	Modified JTT	+
Conforto et al., 2010	RCT (Crossover)	11E, 11C	59.3 ± 1.4 (sub), 64.2 ± 3.7 (supra)	6:5 (sub), 5:6 (supra)	53.1 ± 1.8 (sub), 53.5 ± 2.6 (supra) (days)	6:7 (sub), 7:6 (supra)	RPSS 10 Hz, 1 ms Subsensory (below sensory threshold), suprasensory-(strong paraesthesias)	UL (median nerve) 2 h, 3 days/week for 4 weeks, 12 sessions	–	JTT, pinch strength, FIM	+/–
dos Santos-Fontes et al., 2013	RCT (Parallel Group)	10E, 10C	52.2 ± 11.1E, 59.1 ± 11.1C	5:5E, 6:4C	3.8 ± 4.5E, 3.3 ± 2.1C (years)	6:4E, 7:3C	RPSS 31 Hz Strong paraesthesias	UL (median nerve) 2 h, 7 days/week for 4 weeks, 28 sessions	Sham RPSS and motor training at home	JTT	+
Fleming et al., 2015	RCT (Parallel Group)	16E, 17C	62.3 ± 35-82E, 60.6 ± 24-84C	13:3E, 7:10C	28.9 ± (3–130), 26.6 ± (4–126) (months)	6:10E, 8:9C	SS 10 Hz, 1 ms 3 × sensory threshold	UL (median, radial and ulnar nerves) 2 h, 3 days/week for 4 weeks, 12 sessions	Sham SS paired with/preceding task-specific training	ARAT, FMA-UE, MAL, GAS	+
Hsu et al., 2013	RCT (Parallel Group)	11E:12C	51.1 ± 14.0E, 52.6 ± 13.3C	8:3E, 6:6C	5.8 ± 4.2 E, 9.4 ± 7.1C (months)	8:3E, 6:6C	Thermal stimulation (10 × [15 s + 15 s] × 2 × 2)	LL (distal LE and foot)30 min, 3 days/week for 8 weeks, 24 sessions	Sham/innocuous thermal stimulation with standard rehabilitation	LE-STREAM, mob-STREAM, FAC, BI, PASS, MAS	+/−
Ikuno et al., 2012	RCT (Crossover)	11E (immediate gp), 11C (delayed gp)	68.8 ± 13.9E, 70.1 ± 13.5C	6:5E, 5:6C	91.0 ± 46.5E, 110.3 ± 45.2C (days)	5:6E, 8:3C	PNS 10 Hz, 1 ms Mild paraesthesias	UL (median and ulnar nerve) 1h, 6 days/week for 2 weeks, 12 sessions	Task-oriented training	VAS (level of fatigue), WMFT, BBT, HGS, pinch strength	+/−
In and Cho, 2017	RCT (Parallel Group)	20E, 20C	56.2 ± 10.4E, 56.3 ± 10.2C	11:9E, 12:8C	6.5 ± 2.7E, 6.6 ± 2.5C (months)	10:10E, 11:9C	TENS 100 Hz, 200 ms 2 × sensory threshold	LL (peroneal nerve)30 min, 5 days/ week for 6 weeks, 30 sessions	Sham TENS preceding 30 min sit-to-stand training plus standard therapy	CSI, LL strength, postural-sway distance	+
Klaiput and Kitisomprayoonkul, 2009	RCT (Parallel Group)	10E, 10C	63.0 ± 11.06E, 64.5 ± 10.98C	8:2E, 6:4C	11.9 ± 10.56E, 38.9 ± 54.06C (days)	–	RPSS 10 Hz, 1 ms Strong paraesthesias	UL (median and ulnar nerve) 1 × 2 h session	Sham stimulation	Pinch strength, ARAT	+/–
Lee et al., 2013	RCT (Parallel Group)	16E, 15C	53.31 ± 8.37E, 55.73 ± 8.27C	13:3E, 11:4C	56.94 ± 25.73E, 49.93 ± 29.97C (months)	8:8E, 7:8C	Vibration stimulation 90 Hz, 15 μm	LL (foot-heel, tibialis anterior and achilles tendon)30 min, 3 days/week for 6 weeks, 18 sessions	Sham local vibration stimulus and standard rehabilitation	Postural sway velocity and distance, gait ability	+
Liang et al., 2012	RCT (Parallel Group)	15E, 15C	56.1 ± 11.9E, 59.73 ± 11.6C	12:3E, 7:8C	10.9 ± 5.4E, 13.6 ± 6.4C (days)	9:6E, 8:7C	Thermal stimulation (8 × [30 s+30 s] × 2 × 3)	LL (calf or foot)40 min 5days/week for 6 weeks, 30 sessions	Standard rehabilitation and consults	FMA-LE, MRC LE, BBS, MMAS, MAS, FAC, BI	+
Lin et al., 2014a	RCT (Parallel Group)	14 (MT+MG), 14 (MT)E, 15C	55.79 ± 14.59 MT +MG/ 56.01 ± 12.53 MTE 53.34 ± 10.12C	11:3 (MT+MG); 10:4 (MT)E, 11:4C	22.71 ± 13.62(MT+MG)/ 18.50 ± 11.61(MT)E, 17.80 ± 10.56C (months)	6:8 (MT+MG); 8:6 (MT)E, 7:8C	Afferent stimulation subthreshold (80% of conscious sensory threshold), conscious sensory threshold (100%), above conscious sensory threshold (120%)	UL (hand)1.5 h, 5 days/week for 4 weeks, 20 sessions	Mirror Therapy	FMA, muscle tone (myoton-3), BBT, 10MWT, kinematic parameters, MAL, ABILHAND	+
Lin et al., 2014b	RCT (Parallel Group)	8 (MT+MG)E, 8 (MT)C	56.31 ± 14.79E, 54.97 ± 14.10C	6:2E, 7:1C	18.88 ± 14.78E, 23.38 ± 10.86C (months)	4:4E, 4:4C	SS subthreshold (80% of conscious sensory threshold); conscious sensory threshold (100%); above conscious sensory threshold (120%)	UL (hand)1.5 h, 5days/week for 4 weeks, 20 sessions	Mirror Therapy	MAS, BBT, ARAT, FIM	+/–
Ng et al., 2016	RCT (Parallel Group)	37E, 39C	72.6 ± 97E, 69.3 ± 100C	24:13E, 24:15C	6.1 ± 2.7E, 6.3 ± 2.9C (weeks)	18:19E, 20:19C	TENS parameters (–)	LL (common peroneal and sural nerve) 60 min, 2 days/week for 8 weeks, 16 sessions	Sham stimulation with task-oriented balance training and standard PT and OT	BBS, 6MWT, MRMI, TUGT, SF-36	+/–
Paoloni et al., 2010	RCT (Parallel Group)	22E, 22C	59.5 ± 13.3E, 62.6 ± 9.5C	86.4:13.6%E, 90.9: 9.1%C	1.85 ± 0.59E, 1.86 ± 0.61C (years)	50:50%E, 45.5:54.5%C	Segmental muscle vibration (stimulates 1a spindle afferents) 120 Hz, 10 mm Subthreshold	LL (tibialis anterior and peroneus longus)30 min, 3 days/week over 4 weeks, 12 sessions	Standard PT	Gait time-distance and kinematics	+
Polanowska et al., 2009	RCT (Parallel Group)	20E, 20C	61.6 ± 8.3E, 58.3 ± 12.9C	11:9E; 14:6C	44.4 ± 27.3E, 46.6 ± 26.2C (days)	–	TENS 5 kHz, 100 ms Mild paraesthesias	UL (hand)30 min, 5 days/week for 4 weeks, 20 sessions	Sham stimulation paired with conventional VST	BI, hemineglect severity assessment	–
Seniow et al., 2016	RCT (Parallel Group)	14E, 15C	63.4 ± 7.7E, 60.2 ± 9C	7:7E, 8:7C	40.5 (18.75–105)E, 34.5 (20.25–33.75)C (days)	–	TENS 50 Hz, 300 ms Subthreshold (mild paraesthesias)	UL (hand)30 min, 5 days/week for 3 weeks, 15 sessions	Sham TENS combined with conventional VST	Hemispatial neglect severity assessment	–
Stein et al., 2010	RCT (Parallel Group)	15E, 15C	60.8 ± 14.2E; 66.0 ± 9.0C	46.7:53.3%E: 53.3:46.7%C	5.4 ± 3.6 (0.9–12.6)E, 6.8 ± 4.2 (1.7–13.9)C (years)	53.3%E: 46.7%C	Stochastic resonance stimulation (a) mechanical noise (vibration) bandwidth between/near 0 and 100 Hz, with an amplitude of 0.5- to 1-mm (b) electrical signal bandwidth between/near 0 and 1,000 Hz. Low levels of surface electric current, <150 uA max (50 uA root mean square) Below sensory threshold	UL (upper arm and dorsal forearm)1 h, 3 days/week for 4 weeks, 12 sessions	Sham stimulation and exercise	FMA-UE, WMFT, ARAT, MAS, SIS-16, MAL, RPS, LT (monofilaments), vibration testing, distal proprioception test	–
Sullivan et al., 2012	RCT (Parallel Group)	20E, 18C	61.6 ± (37–88)E, 59.5 ± (41–85)C	13:7E, 14:4C	7.7 ± (1–29)E, 6.6 ± (3–14)C (years)	10:10E, 11:7C	SES 35 Hz, 250 ms Sensory threshold (mild paraesthesias)	UL (forearm) 60 min (2 × 30 min sessions), 5 days/week for 4 weeks, 20 sessions	Sham stimulation (subsensory) during exercise	PTTES, NSA, FMA, AMAT, TS, MAL-14, SIS-16	–
Tyson et al., 2013	RCT (Crossover)	29	64.5 ± 12.6 (28–82)	14:15	–	11:16:2 (bilateral weakness)	TENS 70–130 Hz, 50 us Mild paraesthesias	LL (foot and ankle) 1 × 2 h session	Sham stimulation	DF/PF strength and proprioception detection threshold, FRT, 10MWT	+
Wu et al., 2006	RCT (Crossover)	9	64.5 ± 4.4	5:4	6.5 ± 1.0 (years)	–	PNS 10 Hz, 1 ms Mild paraesthesias	UL (median, ulnar and radial nerves) 1 × 2 h session	No stimulation (sitting and reading)	JTHFT	+
Wu et al., 2010	RCT (Parallel Group)	12E, 11C	59.9 ± 11.4E, 54.3 ± 10.3C	4:8E, 5:6C	10.0 ± 7.3E, 7.2 ± 5.4C (months)	7:5E, 7:4C	Thermal stimulation (10 × [15 s + 15 s] × 2 × 2)	UL (hand and distal arm)30 min, 3 days/week for 8 weeks, 24 sessions	Same thermal stimulation protocol but on LL plus standard therapy	UE-STREAM, ARAT, BI, MAS	+
Yavuzer et al., 2007	RCT (Parallel Group)	15E, 15C	61.9 ± 10.01E, 64.4 ± 9.8C	7:8E, 9:6C	3.5 ± 2.1E, 3.4 ± 2.3C (months)	7:8E, 6:9C	SES 35 Hz, 240 ms Sensory threshold (mild paraesthesias)	LL (common peroneal nerve)30 min, 5 days/4 weeks, 20 sessions	Sham SES and standard PT and OT	Brunnstrom stages, gait time-distance and kinematic characteristics	–

****Abbreviations:*** –, not known; PT/OT, Physio-Occupational Therapy; UL/LL, upper and lower limb. ***Outcome measures:*** AMAT, Arm Motor Ability Test; ARAT, Action Research Arm Test; AS, Ashworth Scale; BBS, Berg Balance Scale; BI, Barthel Index; BBT, Box and Block Test; CSI, Composite Spasticity Index; FAC, Functional Ambulation Category; FIM, Functional Independence Measure; FMA-UE, Fugl Meyer Assessment-Upper Extremity; FRT, Functional Reach/Forward Reaching Test; GAS, Goal Assessment Scale; HGS, Hand Grip Strength; JTHFT/JTT, Jebsen Hand Function Test; LE-STREAM/Mob-STREAM, Lower Extremity/mobility subscale of Stroke Rehabilitation Assessment of Movement; MAL, Motor Activity Log; MAS, Modified Ashworth Scale; MMAS, Modified Motor Assessment Scale; MRC-LE scale, Medical Research Council scale for Lower Extremity; MRMI, Modified Rivermead Mobility Index; NSA, Nottingham Sensory Assessment; PASS, Postural Assessment Scale for Stroke; PTTES, Perceptual Threshold Test-Electrical Stimulation; RPS, Reaching Performance Scale; SF-36, short form general health questionnaire; TS, tardieu scale; TUGT, Timed Up and Go Test; UE-STREAM, Upper Extremity subscale of Stroke Rehabilitation Assessment of Movement; VAS, visual analog scale; WMFT, Wolf Motor Function Test; 10MWT/6MWT, 10/6 Meter Walk Test. ***Interventions:*** MNS, Median Nerve Stimulation; PNS, Peripheral Nerve Stimulation; RPSS, Repetitive Peripheral Nerve Stimulation; SS, somatosensory stimulation; SES, Sensory amplitude Electrical Stimulation; SWT, Short Wave Therapy; TENS, Transcutaneous Electrical Nerve Stimulation; TS, thermal stimulation; VST, visual scanning training*.

*Training duration and controls:* Training duration varied from 20 to 180 min, 1 to 7 sessions/week over a period of 1–12 weeks, with the number of sessions ranging from 1 to 30. Fifteen studies used sham stimulation without current delivered/turned off as the control (Cambier et al., 2003; Yavuzer et al., 2007; Klaiput and Kitisomprayoonkul, 2009; Polanowska et al., 2009; Stein et al., 2010; Sullivan et al., 2012; dos Santos-Fontes et al., 2013; Lee et al., 2013; Tyson et al., 2013; Fleming et al., 2015; Carrico et al., 2016a,b; Ng et al., 2016; Seniow et al., 2016; In and Cho, 2017). Five studies used conventional rehabilitation (Chen et al., 2005, 2011; Paoloni et al., 2010; Ikuno et al., 2012; Liang et al., 2012), while Conforto used subthreshold low-frequency stimulation (Conforto et al., 2007) and Conforto did not use a control (Conforto et al., 2010). Three studies did not deliver any stimulation (Conforto et al., 2002; Wu et al., 2006; Celnik et al., 2007). Wu used the same thermal stimulation protocol but on the lower limb not upper limb (Wu et al., 2010), Hsu an innocuous thermal stimulation protocol (Hsu et al., 2013), and Lin used mirror therapy (Lin et al., 2014a,b).

*Outcome measures:* A broad range of measures were used, however the most commonly assessed functional measures were ARAT (Klaiput and Kitisomprayoonkul, 2009; Stein et al., 2010; Wu et al., 2010; Lin et al., 2014b; Fleming et al., 2015; Carrico et al., 2016a,b), JTHFT (Wu et al., 2006; Celnik et al., 2007; Conforto et al., 2007, 2010; dos Santos-Fontes et al., 2013), WMFT (Stein et al., 2010; Ikuno et al., 2012; Carrico et al., 2016a,b) and Barthel Index (Polanowska et al., 2009; Wu et al., 2010; Liang et al., 2012; Hsu et al., 2013). While the most commonly used impairment-based measures were FMA (Cambier et al., 2003; Stein et al., 2010; Chen et al., 2011; Liang et al., 2012; Sullivan et al., 2012; Lin et al., 2014a; Fleming et al., 2015; Carrico et al., 2016a,b), modified Ashworth Scale (Cambier et al., 2003; Chen et al., 2005, 2011; Stein et al., 2010; Wu et al., 2010; Hsu et al., 2013; Lin et al., 2014b) and Brunstromm Stages (Chen et al., 2005; Yavuzer et al., 2007; Paoloni et al., 2010).

##### Active sensory training interventions

Four studies delivered sensory discrimination training (see Table 5). All studies showed positive effects with three upper limb studies indicating improvements on sensation, arm and hand function as well as gait (Byl et al., 2003; Carey et al., 2011; de Diego et al., 2013), while the lower limb study highlighted changes in postural sway (Morioka and Yagi, 2003). Two studies also showed positive results, one lower limb study delivered sensory education and retraining with improvements found on sensation, gait and mobility (Lynch et al., 2007). Another upper limb study investigated Perfetti's method (a cognitive sensory motor training approach) and showed no effect on arm and hand function or mobility (Chanubol et al., 2012).

**Table 5:** Active sensory training study characteristics.

**Author, year**	**Study design**	**Sample size**	**Age (years)**	**Gender (M:F)**	**Stroke duration (time since stroke) mean (SD)**	**Side of stroke (affected side)**	**Intervention**	**Target duration**	**Control condition**	**Outcome measures**	**Direction of effects (+ positive, – negative, +/- both)**
Byl et al., 2003	RCT (Crossover)	8E, 10C	69.0, 58.5	5:3, 7:3	4.5, 4.8	5:3, 5:5	Sensory discrimination training (and mental imagery, mirror and functional practice at home)	UL 1.5 h/week for 4 weeks, 4 sessions [and HEP CIMT 7 h + 15–90 min functional practice]	–	Sensory discrimination (kinesthesia, graphesthesia, stereognosis), PPT, WMFT, Cal-FCP, UL/LL strength and ROM, gait speed	+
Carey et al., 2011	RCT (Parallel/Crossover)	25E, 25C	61.08 ± 14.38E, 60.96 ± 11.17C	17:8E, 20:5C	32.57 (12.22–111.22)E, 51.86 (20.57–72.53)C (weeks)	40:60%E, 44:56%C	Somatosensory discrimination training (texture discrimination, limb position sense, and tactile object recognition)	UL 60 min, 3 sessions/week for ~4 weeks, 10 sessions	Exposure to sensory stimuli	Composite index of functional somatosensory discrimination capacity: FMT, WPST, fTORT	+
Chanubol et al., 2012	RCT (Parallel Group)	20E, 20C	63.2 ± 10.1	9:11E, 11:9C	-	1:19E, 1:19C	Perfetti's method (cognitive sensory motor training therapy- perception tasks: sensing and discriminating limb positions)	UL 30 min, 5 times/week for 4 weeks, 20 sessions	Standard OT	ARAT, BI, BBT	–
de Diego et al., 2013	RCT (Parallel Group)	12E, 9C	61.9 ± 9.7E, 60.6 ± 15.6C	-	44.7 ± 24.5E, 60.7 ± 58.2C (months)	–	Sensory stimulation and functional activity training (targeting tactile stimulation, mental imagery and practice of ADL's at home)	UL 1 h, 2 days/week over 8 weeks, 16 sessions [and HEP 30 min/day over 8 weeks total 28 h]	Standard rehabilitation	FMA-UE, MAL, SIS-16, sensory discrimination battery: tactile sensibility (monofilaments), proprioceptive sensibility (passive ROM), consistency and weight discrimination (ordering consistency and weight of objects)	+
Lynch et al., 2007	RCT (Parallel Group)	10E, 11C	61.0 ± 15.8, (21–77)E, 62.0 ± 12.3, (38–82)C	7:3E, 9:2C	48.7 ± 31.1 (19–122)E, 47.8 ± 27.7 (13–112)C (days)	5:5E, 8:3C	Sensory retraining (education, detection, localization, discrimination and proprioception)	LL(foot) 30 min, 10 sessions over 2 weeks	Sham relaxation and standard PT	LT monofilaments, distal proprioception test, BBS, gait time and Iowa	+/–
Morioka and Yagi, 2003	RCT (Parallel Group)	12E, 14C	62.6 ± 13.3 (51–79)E, 61.3 ± 11.0 (56–73)C	9:3E, 8:6C	65.4 ± 18.6 (36–106)E, 61.9 ± 20.8 (31–111)C (days)	6:6E, 5:9C	Perceptual learning exercises (hardness discrimination)	LL 10 trials/session, 10 days over 2 weeks	Standard PT/OT (no perceptual learning exercise)	SBT (postural sway)	+/–

****Abbreviations:*** –, not known; UL/LL, upper and lower limb; ***Outcome measures:*** Cal-FCP, California Functional Capacity Evaluations; FMT, Fabric Matching Test; fTORT, Tactile Object Recognition Test; PPT, Purdue Pegboard Test; SBT, Stabilometer Balance Test; WMFT, Wolf Motor Function Test; WPST, Wrist Position Sense Test; ***Interventions:*** ADL's, activities of daily living; CIMT, constraint induced movement therapy; HEP, home exercise program*.

*Training durations and controls:* Training duration varied from 30 to 90 min, 1 to 5 days/week over a period of 2–8 weeks, with the number of sessions ranging from 4 to 20. Three studies used standard rehabilitation as the control (Morioka and Yagi, 2003; Chanubol et al., 2012; de Diego et al., 2013), while Lynch used sham relaxation in addition to standard rehabilitation (Lynch et al., 2007). Carey used a comparative control exposure to sensory stimuli (Carey et al., 2011) and Byl did not use a control (Byl et al., 2003).

*Outcomes measures:* Most common functional outcomes measures included ARAT (Chanubol et al., 2012), WMFT (Byl et al., 2003), MAL and SIS-16 (de Diego et al., 2013). The most common impairment-based measures were FMA (de Diego et al., 2013) and a varied battery of sensory tests including discrimination (texture, weight, consistency), tactile sensibility (Semmes-Weinstein monofilaments) and object recognition and proprioception (wrist position sense test) (Byl et al., 2003; Lynch et al., 2007; Carey et al., 2011; de Diego et al., 2013).

##### Hybrid sensory training interventions

Three studies did not fit within the active or passive group alone and were considered hybrid (see Table 6). One study focused on sensory-based and stabilizer-based trunk feedback and showed no significant effects on arm function (Thielman, 2010). Another study delivered one of three virtual-reality based rehabilitation configurations: vision-based tracking, haptic feedback (primary interest) or a passive exoskeleton and indicated no significant between-group differences on arm and hand function, spasticity or mobility (Cameirão et al., 2012). While the third study delivered four types of somatosensory stimulation (no stimulation, vibration, and light and rough touches) with improvements on arm and hand function, particularly following vibration (Sim et al., 2015).

**Table 6:** Hybrid sensory training study characteristics.

**Author, year**	**Study design**	**Sample size**	**Age (years)**	**Gender (M:F)**	**Stroke duration (time since stroke) mean (SD)**	**Side of stroke (affected side)**	**Intervention (active)/(passive-frequency, pulse length, intensity)**	**Target duration**	**Control condition**	**Outcome measures**	**Direction of effects (+ positive, – negative, +/– both)**
(Cameirão et al., 2012)	RCT (Parallel Group)	16 (RGS-vision-based tracking system alone), 14 (RGS exoskeleton), 14 (RGS haptics)	68.7 ± 10.9 RGS, 59.4 ± 9.7 RGS-E, 59.9 ± 13.0 RGS-H	9:7 RGS, 9:5 RGS-E, 7:7 RGS-H	1649 ± 300 RGS, 1598 ± 230 RGS-E, 1334 ± 297 RGS-H (days)	6:10 RGS, 4:10 RGS-E, 6:8 RGS-H	Rehabilitation gaming system (configurations: vision-based tracking, passive exoskeleton or haptics)	UL 35 min/day, 5 days/week for 4 weeks, 20 sessions	No control	BI, MI-UE, MAS, FMA-UE, CAHAI, 9-HPT, BBT	+/–
(Sim et al., 2015)	RCT (Crossover)	11	60.36 ± 12.39	7:4	12.64 ± 11.02 (months)	5:6	SS (no stimulation, vibration, light and rough touches)	UL 1 × 5 min session	No stimulation	BBT, JTHFT, HGS, FRT	+
(Thielman, 2010)	RCT (Parallel Group)	8E (sensor), 8C (stabilizer)	62.9 ± 6.5 sensor, 63 ± 9.2 stabilizer	6:2 sensor, 4:4 stabilizer	26.5 sensor, 22.75 stabilizer (months)	4:4 sensor, 4:4 stabilizer	Auditory feedback sensor to pressure or stabilizer feedback (trunk restraint)	Trunk 40–45 min, 2-3 days/week for 4–6 weeks, 12 sessions	No control	RPS, FMA-UE, AROM, HGS, WMFT, MAL	+/–

****Abbreviations:*** UL/LL, upper and lower limb. ***Outcomes:*** CAHAI, Chedoke arm and hand activity inventory; MI, Motricity Index; 9-HPT, nine-hole peg test. ***Interventions:*** SS, somatosensory stimulation*.

*Training duration and controls:* Training duration varied from 5 to 45 min, 1 to 5 sessions/week over a period of 4–6 weeks, with the number of sessions ranging from 1 to 20. Two studies used no controls and were comparative studies (Thielman, 2010; Cameirão et al., 2012), while one used no stimulation as the control condition (Sim et al., 2015).

*Outcome measures:* Most commonly used functional outcomes measures were BBT (Cameirão et al., 2012; Sim et al., 2015) and WMFT (Thielman, 2010). While impairment-based measures included FMA (Thielman, 2010; Cameirão et al., 2012), modified Ashworth scale (Cameirão et al., 2012) and range of motion and strength (Thielman, 2010; Sim et al., 2015).

#### Risk of Bias

**Figure 2:**
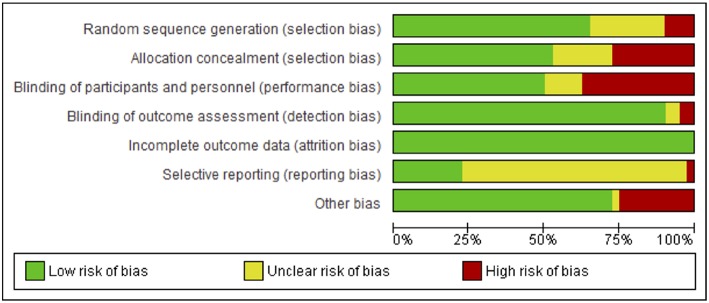
Assessment of risk of bias presented as percentages across all included studies.

**Figure 3:**
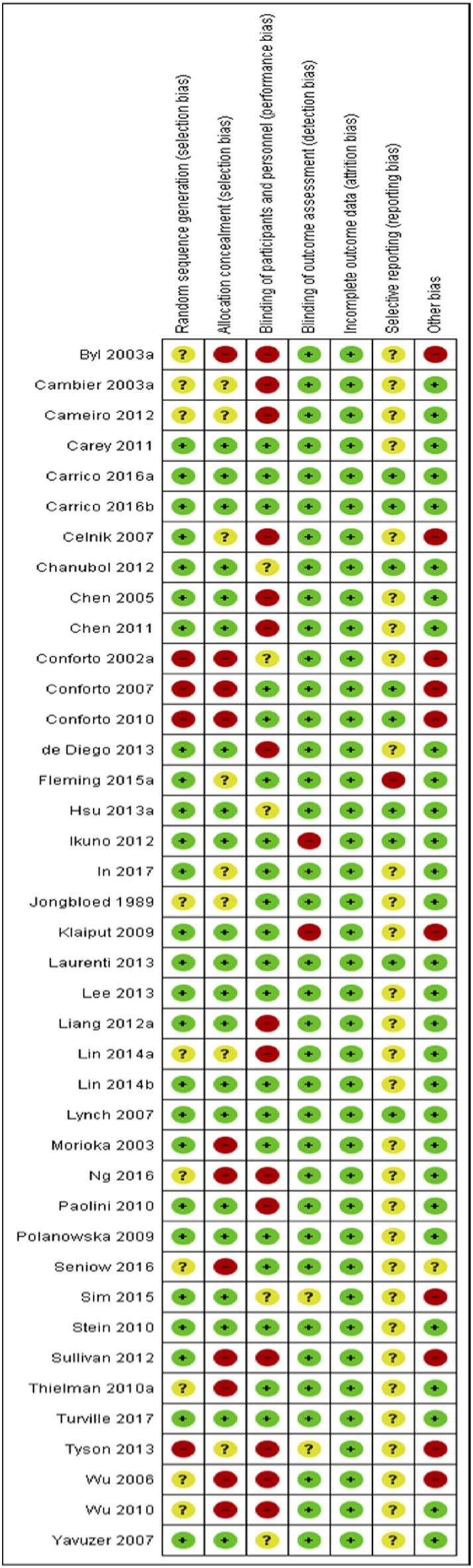
Risk of bias summary for each included study.

Risks to methodological quality were prominent in the assessment of selection, performance and reporting biases. An assessment summary is presented in Figure 2, and details for each study are provided in Figure 3. High risk of selection biases were most frequent within the domains of performance biases from a lack of participant and/or personnel blinding, however this is a common, and often unavoidable part of physiotherapy and occupational therapy intervention research designs. Further high risk biases were found within the domains of selection bias including inadequate random sequence generation and allocation concealment as well as other biases due to small sample size limiting generalization to the wider population, single session interventions and lack of follow-up testing (reducing the ability to extrapolate results from repeated sessions and increasing the difficulty to understand findings beyond the study procedures). Further, we noted potential biases of control conditions including sham stimulation which may cause central afferent input affecting cortical reorganization and study outcomes, difficulty putting on/setting up equipment (electrode glove) compromising practice, lack of rigorous procedures to monitor subject compliance at home and during passive stimulation, absence of an independent intervention group to delineate effects of standard rehabilitation, potential carryover effects in crossover and study design limited by using only one group or no control group. There was an unclear risk of bias within reporting biases including selective reporting of results due to lack of, or unclear, protocol registration and reporting of randomized controlled trial study designs, and again within the domain of selection biases (random sequence generation and allocation concealment) and performance bias (blinding of participants and personnel). However, detection and attrition biases were generally well reported and of low risk.

## Discussion

### Summary of Main Findings

The purpose of this review was to evaluate the body of literature around sensory-based interventions to improve sensation and/or sensorimotor function of individuals following stroke. This is an important question as sensory-based interventions have largely been overlooked despite the indication that they are likely to form a critical component of stroke recovery. This review found 38 full-text manuscripts that investigated sensory-based interventions in people with stroke. We categorized these interventions into passive, active or hybrid. The key findings from the meta-analyses suggest that there is some evidence to support the use of passive sensory techniques with improved outcomes following thermal stimulation, pneumatic compression and peripheral nerve stimulation. The data for active sensory training was limited with most findings reported narratively, many highlighting positive activity-based outcomes. The large number of techniques reviewed did show promise in addressing sensation and sensorimotor function following stroke however at this stage we continue to not have adequate high quality trials to be able to make clear recommendations regarding the use of passive and active interventions.

Findings continue to suggest passive sensory training may enhance the effects of current task-oriented training and may be a useful adjunct when combined with standard rehabilitation (Schabrun and Hillier, 2009; Doyle et al., 2010). Only two studies reported no effect, one delivering stochastic resonance stimulation and the other sensory amplitude electrical stimulation (Stein et al., 2010; Sullivan et al., 2012). The limited high-quality research for active sensory training continues to neither affirm or negate its use, suggesting it may be effective as a supplemental training program and applied with careful clinical reasoning and measurement of individual effects. Two studies showed improvements following sensory discrimination training (Byl et al., 2003; Carey et al., 2011), with only one study exploring Perfetti's method showing no effect when compared to usual care (Chanubol et al., 2012). Findings from hybrid studies suggest somatosensory stimulation may be beneficial with positive effects found in a single study for vibration stimulation (Sim et al., 2015), however less clear effects were found for somatosensory-based feedback and virtual reality-based haptics (Thielman, 2010; Cameirão et al., 2012). Compared to previous reviews (Schabrun and Hillier, 2009; Doyle et al., 2010), we have found a greater number of studies addressing a broader range of interventions and outcome measures, however the general findings have not changed significantly and similar issues need to be addressed. The lack of sufficient literature to perform meta-analyses and insignificant effect sizes continue to mean it is not possible to determine the effectiveness of sensory retraining, particularly for the active group (Schabrun and Hillier, 2009; Doyle et al., 2010).

Implications for practice: Health professionals may use this evidence to guide clinical decision-making surrounding sensory training following stroke. Few studies mentioned (or evaluated) adverse effects: clinicians need to be conscious of monitoring these effects when using any sensory-based interventions. Careful consideration must also be taken by therapists regarding the suitability of sensory training for the individual prior to clinical application to improve individual functional outcomes particularly when active participation is required.

Implications for research: The significant number of individuals that continue to experience sensory deficits following stroke and the potential benefits of sensory training identified in this review indicate further research is essential. High-quality randomized controlled trials with high statistical power and rigorous methods including consistent and homogenous outcome measures are required to support or refute the effectiveness of sensory training, particularly active sensory training following stroke. Sufficient reporting of the type of intervention and training parameters are required to allow replication of the sensory training protocol.

### Limitations

All studies included were randomized controlled trials which are considered the ‘gold standard' when determining treatment effectiveness as this robust methodological design minimizes the effects of bias. Methodological quality was reasonable across most studies, however, there were areas where methodological rigor was notably lacking introducing the potential for bias and reducing confidence in the findings. The results may have been influenced by widespread lack of blinding of participants and personnel with the potential for performance biases, lack of concealment with the potential for selection biases and the potential for other biases with seven of the included studies implementing a single treatment session. These were included as they met the selection criteria, however the therapeutic effect of a single session has heightened the risk of biases as these data cannot be extrapolated to results from repeated sessions as would occur in a clinical setting. Of the 38 randomized controlled trials, only nine studies justified the selected sample size while 23 of these sample sizes were relatively small and six only mentioned the total sample size limiting capacity to observe significant effects. This may mean the insufficient evidence in this review may be due to poor statistical power rather than ineffective intervention. Reliability and validity of measures used were strong, however, passive training studies predominantly used measures relating to motor activity (ARAT, WMFT), while active training focused on measures at the impairment level (tactile sensibility, sensory discrimination). The impairment-based measures may be more sensitive to detecting change and any changes are likely to be of a smaller magnitude and not reach statistical significance as easily, while changes in function are generally larger and may be the results of net improvements in sensation, proprioception and motor function rather than one single component (Schabrun and Hillier, 2009; Doyle et al., 2010). This has particularly impacted on forming conclusions in the active group. Seven studies focused on balance and postural control, these were excluded as they were considered to manipulate multi-modal sensory input (particularly vision) rather than augment which was the primary focus of this review. Most studies only reported selective outcomes increasing the potential risk for reporting biases. Most active and passive studies reported standard rehabilitation or sham stimulation as the comparator, however again these were poorly defined which may have resulted in greater variability between studies particularly in the active group. In addition, the high heterogeneity between types of intervention, intervention parameters and outcomes measures made it difficult to produce clear comparisons in the meta-analyses and prevented the ability to perform subgroup analyses.

## Conclusions

This review sought to provide an updated review investigating the effects of sensory training protocols on somatosensory function following stroke. Although a greater number of studies have been published since the previous reviews in 2009 and 2010 (Schabrun and Hillier, 2009; Doyle et al., 2010) only a small number of these studies were of high quality with a greater focus on passive sensory training than active. Findings indicate there is some evidence to support the use of passive sensory techniques and while data for active sensory training is limited it does show promise in improving sensation and sensorimotor function following stroke. The ability of this review to form sound conclusions and develop clear recommendations regarding sensory training in stroke rehabilitation continues to be affected by the limited high-quality studies and the diverse range of interventions and outcome measures.

## Clinical Messages

- Passive sensory interventions may assist in improving activity following stroke.
- Evidence for active sensory training continues to be limited by research design, small sample size and heterogeneous outcome measures.
- Further high-quality research is required to determine the effectiveness of sensory training in stroke rehabilitation, particularly active-based therapy.

## Data Availability

All datasets generated for this study are included in the manuscript and/or the Supplementary Files.

## Author Contributions

All authors listed have made a substantial, direct and intellectual contribution to the work, and approved it for publication.

### Conflict of Interest Statement

The authors declare that the research was conducted in the absence of any commercial or financial relationships that could be construed as a potential conflict of interest.
