# Supplementary material for: Does Sensory Retraining Improve Sensation and Sensorimotor Function Following Stroke: A Systematic Review and Meta-Analysis
Source: Front Neurosci. 2019 Apr 30;13:402. doi: 10.3389/fnins.2019.00402 (PMC6503047; doi:10.3389/fnins.2019.00402)
Supplement: Supplementary file 1 [file Data_Sheet_1.docx]

**Supplementary Materials**

**Appendix 1**

**Table 7** Search strategy in Ovid Medline database

| 1. cerebrovascular disorders/ or exp basal ganglia cerebrovascular disease/ or exp brain ischemia/ or exp carotid artery diseases/ or exp intracranial arterial diseases/ or exp "intracranial embolism and thrombosis"/ or exp intracranial hemorrhages/ or stroke/ or exp brain infarction/ or vasospasm, intracranial/ or vertebral artery dissection/ 2. (stroke or poststroke or post-stroke or following stroke or cerebrovasc$ or cerebral vasc$ or cerebral vas$ accident or cerebrovascular accident brain vasc$ or cerebral vasc$ or cva$ or apoplex$ or SAH).tw. 3. ((brain$ or cerebr$ or cerebell$ or intracran$ or intracerebral) adj5 (isch?emi$ or infarct$ or thrombo$ or emboli$ or occlus$)).tw. 4. ((brain$ or cerebr$ or cerebell$ or intracerebral or intracranial or subarachnoid) adj5 (haemorrhage$ or hemorrhage$ or haematoma$ or hematoma$ or bleed$)).tw. 5. hemiplegia/ or exp paresis/ 6. (hemipleg$ or hemipar$ or paresis or paretic).tw. 7. 1 or 2 or 3 or 4 or 5 or 6 8. sensation/ or exp proprioception/ or exp kinesthesis/ or exp touch/or exp touch perception/ or exp sensation disorders/ or exp somatosensory disorders/ or exp stereognosis/ or exp agnosia/ or exp psychomotor disorders/or exp electric stimulation/ 9. (sensation or sensory or somatosensory or propriocept$ or kinesthesi$ or touch or stereognosis or tactile or two-point discrimination or position sense).tw. 10. (sensory train$ or sensory retrain$ or sensory education or sensory re-education or sensory reeducation or sensory rehabilitat$ or sensory practice or sensory treatment$ or sensory awareness or sensory movement$ or sensory intervention$ or sensory discrimin$).tw. 11. (cutaneous stimulat$ or electrical stimulat$ or electric stimulat$ or stimulat$ therap$ or afferent stimulat$ or sensory stimulat$ or somatosens$ stimulat$).tw. 12. 8 or 9 or 10 or 11 13. 7 and 12 14. limit 14 to human |
| --- |

**Appendix 2** Forest plot illustrating the effect size (95% CIs) for sensory training compared to usual care


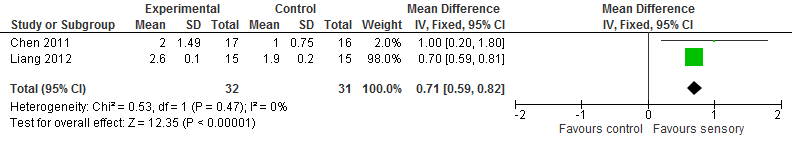


**Figure 4** Functional Ambulation Category (FAC)


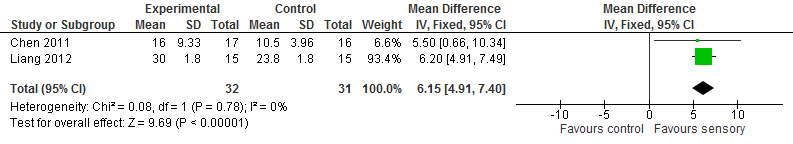


**Figure 5** Motor Assessment Scale (MAS)


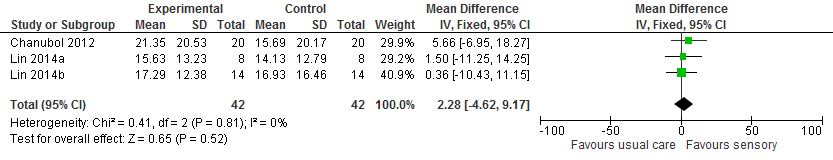


**Figure 6** Box and Block Test (BBT)


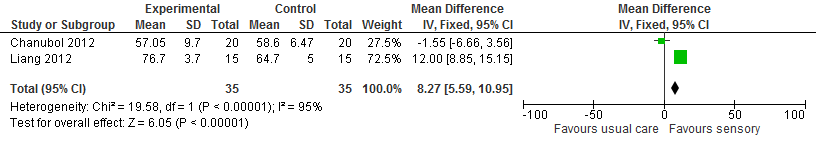


**Figure 7** Barthel Index (BI)


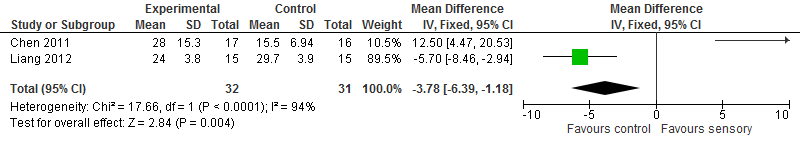


**Figure 8** Berg Balance Scale (BBS)


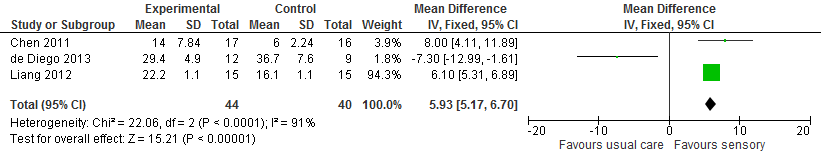


**Figure 9** Fugl-Meyer Assessment (FMA)

**Appendix 3** Forest plot illustrating the effect size (95% CIs) for sensory training compared to sham


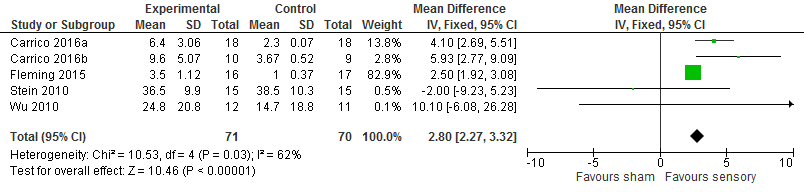


**Figure 10** Action Research Arm Test (ARAT)


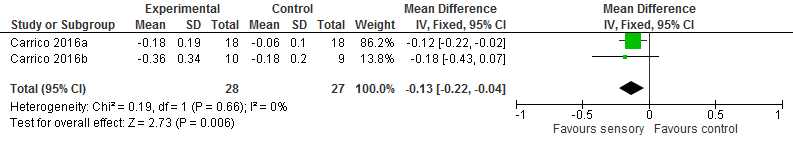


**Figure 11** Wolf Motor Function Test (WMFT)


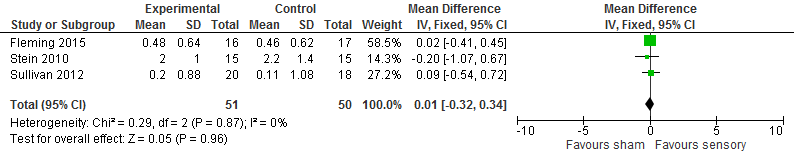


**Figure 12** Motor Activity Log (MAL)


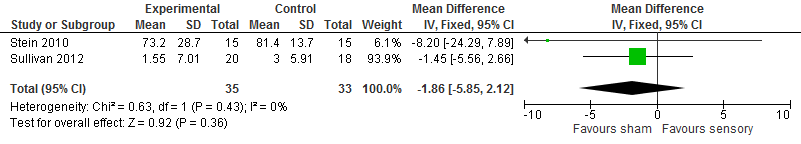


**Figure 13** Stroke Impact Scale (SIS)


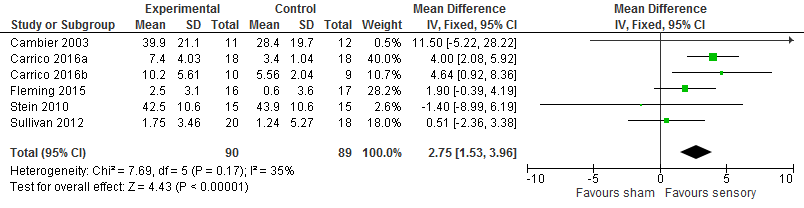


**Figure 14** Fugl-Meyer Assessment (FMA)


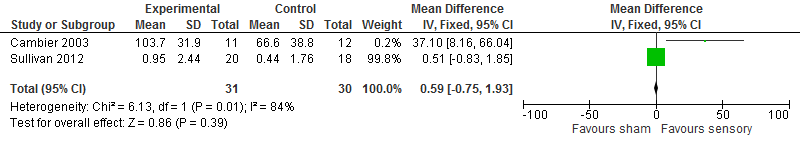


**Figure 15** Nottingham Sensory Assessment (NSA)

**Appendix 4**

**Table 8** Studies awaiting classification/consideration

| References |
| --- |
| 1. Bonan I, Leblong E, Damphousse M, Tassel-Tonche S, Yelnik A. Effect of sensory stimulations in improving balance after stroke. Annals of Physical and Rehabilitation Medicine. 2014; 1:165. |
| 1. Carey L, Matyas T, Walker J, Macdonell R. Sense: Study of the effectiveness of neurorehabilitation on sensation; individual patient characteristics that predict favourable outcomes. International Journal of Stroke 2010; 5:26. |
| 1. Clarke J, Lynch M, Dilworth C. Portable electrical stimulation early following stroke: Risks and recovery. International Journal of Stroke. 2013; 2:11. |
| 1. Cupti F, Rocco IS, Puccini C, Garcia MAC, Ribeiro RS, Andre C, et al. Comparison between two types of electrical stimulation in the treatment of the upper extremity in persons with a stroke. 2011. |
| 1. Ellis DW, Rader MA. Structured sensory stimulation. Physical Medicine and Rehabilitation: State of the Art Reviews. 1990; 4(3):465-77. |
| 1. Exell T, Meadmore K, Freeman C, Kutlu M, Hughes AM, Burridge J. Goal-orientated functional rehabilitation using electrical stimulation and iterative learning control for motor recovery in the upper extremity post-stroke. International Journal of Stroke 2013; 3:29. |
| 1. Ghanjal A, Motaqhey M, Hafezi R, Ghasemi M. The effect of sensory retraining on upper limb functional recovery in patients with ischemic stroke. Journal of Zanjan University of Medical Sciences and Health Services 2016; 24(103):10-9. |
| 1. Gobert D, Merring C, Dugan K. Somatosensory stimulation combined with moderate intensity therapeutic exercise significantly improves motor function in chronic stroke survivors. Stroke. 2013; 44(2). |
| 1. Ikuno K, Kitabeppu S, Nagino K, Morimoto S, Matsuo A, Shomoto K. Effects of task-oriented training with somatosensory stimulation on paretic-hand function in patients with subacute stroke: A pilot randomized controlled trial. 2011. |
| 1. Johansson BB. Has sensory stimulation a role in stroke rehabilitation? Scandinavian Journal of Rehabilitation Medicine. 1993; (Suppl 29):87-96. |
| 1. Jongbloed L, Stacey S, Brighton C. Stroke rehabilitation: sensorimotor integrative treatment versus functional treatment. American Journal of Occupational Therapy. 1989; 43(6):391-7. |
| 1. Kim Y, Yi CH, Lee YH, Jeon HS, Chung Y. Immediate effects of dermatomal electrical stimulation on task-oriented movements in patients with chronic hemiplegia. Journal of Physical Therapy Science. 2013; 25(1):89-91. |
| 1. Koenig LS, Christova M, Schwenker K, Bartsch H, Luthringshausen G, Gallasch E, et al. Modulation of cortical plasticity by whole-hand electrical stimulation in attempt to improve hand motor functions after stroke. Journal of the Neurological Sciences. 2013; 1:547. |
| 1. Koesler IBM, Dafotakis M, Ameli M, Fink GR, Nowak DA. Electrical somatosensory stimulation improves movement kinematics of the affected hand following stroke. Journal of Neurology, Neurosurgery and Psychiatry. 2009; 80(6):614-9. |
| 1. Kumar V, Kubera P, Joshua AM, Misri ZK, Suresh BV, Chakrapani M. Effects of task oriented exercises with altered sensory input on functional mobility in chronic stroke - A randomized controlled trial. Cerebrovascular Diseases 2013; 1:14. |
| 1. Kuys SS, Clarke J, Dilworth C, Lynch M. Peroneal electrical stimulation: Application early following stroke. International Journal of Stroke. 2013; 1:29-30. |
| 1. Liu W, Mukherjee M, Kim SH, Liu H, Natarajan P, Agah A. Developing a sensory-enhanced robot-aided motor training programme. International Journal of Mechatronics and Automation 2011; 1(3-4):236-43. |
| 1. Sawan S, AbdAllah F, Mohamed N, Hegazy MM, Gohar YS. The effect of surface spinal electric sensory stimulation on functional outcome in spastic stroke patients. 2011. |
| 1. Seo N, Downey R, Dellenbach B, Imburgia R, Lauer A, Ramakrishnan V, et al. TheraBracelet sensory stimulation to enhance hand functional recovery post stroke. Archives of Physical Medicine & Rehabilitation. 2017. |
| 1. Tang W, Yang B. Influence of sensory function training on motor function of cerebral apoplexy patients complicated with hemiplegia. Chinese Nursing Research. 2009; 23(7C):1905-6. |
| 1. Tekeolu Y, Adak B, Goksoy T. Effect of transcutaneous electrical nerve stimulation (TENS) on Barthel Activities of Daily Living (ADL) Index score following stroke. 12. 1998; 4(277-280). |
| 1. Torriani C, Mota EP de O, Moreira Sales AL, Ricci M, Nishida P, Marques L, et al. Effect of foot motor and sensorial stimulation hemiparetic in stroke patients. 2008. |
| 1. Turville M, Carey LM, Matyas TA, Blennerhassett J. Change in functional arm use is associated with somatosensory skills after sensory retraining post stroke. American Journal of Occupational Therapy 2017; 71(3):1-9. |
| 1. Weinberg J, Diller L, Gordon WA, Gerstman LJ, Lieberman A, Lakin P, et al. Training sensory awareness and spatial organization in people with right brain damage. Archives of Physical Medicine & Rehabilitation 1979; 60(11):491-6. |
| 1. Wongphaet P, Chira-Adisai W, Chanubol P, Tantisupawongse D. Effects of cognitive sensory motor training in subacute stroke. Clinical Neurophysiology. 2009. |
| 1. Wu CYH, P. C.; Lin, K. C.; Huang, W. L. Efficacy of mirror therapy combined with afferent stimulation on motor impairment, motor function, and daily function in chronic stroke: A randomized controlled trial. American Heart Association's Quality of Care and Outcomes Research in Cardiovascular Disease and Stroke. 2013; 6(3(Suppl. 1)). |
| 1. Wu C, Lin K, Huang P, Liing R. Hybrid Approach to Mirror Therapy and Somatosensory Stimulation for Rehabilitating Movement and Function Post Stroke. Archives of Physical Medicine & Rehabilitation. 2013. |
| 1. Yamaguchi T, Ikuno K, Fuchigami K, Koyama S, Fujikawa K, Kobayashi H, et al. Pedaling exercise combined with sensory electrical stimulation improves gait performance in subacute stroke patients: A multicenter, sham-controlled randomized controlled trial. Physiotherapy. 2015. |

**Appendix 5**

**Table 9** Study Exclusions

| References | Reason |
| --- | --- |
| 1. Abdollahi F, Case Lazarro ED, Listenberger M, Kenyon RV, Kovic M, Bogey RA, et al. Error augmentation enhancing arm recovery in individuals with chronic stroke: A randomized crossover design. Neurorehabilitation and Neural Repair. 2014; 28(2):120-8. | Intervention: visual kinematic biofeedback |
| 1. Altschuler EL, Wisdom SB, Stone L, Foster C, Galasko D, Llewellyn DM, et al. Rehabilitation of hemiparesis after stroke with a mirror. Lancet. 1999; 353(9169):2035-6. | Intervention: mirror therapy |
| 1. Au-Yeung SS. Treatment of upper extremity paresis using transcutaneous electrical stimulation during acute stroke: Hong Kong Polytechnic University 2006. | Intervention: acupoint electrical stimulation |
| 1. Au-Yeung SS, Hui-Chan CW. Electrical acupoint stimulation of the affected arm in acute stroke: a placebo-controlled randomized clinical trial. Clinical Rehabilitation. 2014; 28(2):149-58. | Intervention: acupoint electrical stimulation |
| 1. Barker RN, Brauer SG, Carson RG. Training of reaching in stroke survivors with severe and chronic upper limb paresis using a novel nonrobotic device: a randomized clinical trial. Stroke. 2008; 39(6):1800-7. | Intervention: stimulation elicits motor contraction |
| 1. Bayouk J, Boucher JP, Leroux A. Balance training following stroke: effects of task-oriented exercises with and without altered sensory input. International Journal of Rehabilitation Research. 2006; 29(1):51-9. | Intervention: manipulating sensory input |
| 1. Beaulieu L, Massé-Alariea H, Camiré-Berniera S, Ribot-Ciscarb E, Schneidera C. After-effects of peripheral neurostimulation on brain plasticity and ankle function in chronic stroke: The role of afferents recruited. Clinical Neurophysiology. 2017; 47:275-91. | Intervention: NMES |
| 1. Ben-Shabat E, Carey LM, Matyas TA, Brotchie PR. A brain activation study of limb position sense in stroke affected individuals with and without sensory training, and in healthy aged. Internal Medicine Journal. 2006; 36(A14). | Study design: not an RCT |
| 1. Bi S. Treatment of upper limb paresis by transcutaneous electrical nerve stimulation and task-related training during chronic stroke: Hong Kong Polytechnic University; 2008. | Intervention: acupoint electrical stimulation |
| 1. Byl NN, Pitsch EA, Abrams GM. Functional outcomes can vary by dose: Learning-based sensorimotor training for patients’ stable post stroke. Neurorehabilitation and Neural Repair. 2008; 22(5):494-504. | Study design: not an RCT |
| 1. Calabro RS, De Cola MC, Leo A, Reitano S, Balleta T, Trombetta G, et al. Robotic neurorehabilitation in patients with chronic stroke: psychological well-being beyond motor improvement. International Journal of Rehabilitation Research. 2015; 38:219-25. | Study design: not an RCT |
| 1. Carey LM, Abbott DF, Lamp G, Puce A, Seitz RJ, Donnan GA. Same Intervention-Different Reorganization: The impact of lesion location on training-facilitated somatosensory recovery after stroke. Neurorehabilitation and Neural Repair. 2016; 30(10):988-1000. | Study design: not an RCT |
| 1. Carey LM, Matyas TA. Training of somatosensory discrimination after stroke: facilitation of stimulus generalization. American Journal of Physical Medicine and Rehabilitation. 2005; 84(6):428-42. | Study design: not an RCT |
| 1. Carey LM, Matyas TA. Response to Four weeks (10 sessions) of individual sensory discrimination training produced clinically important changes in upper limb sensation after stroke. Australian Occupational Therapy Journal 2012; 59:168-9. | Summary of an included RCT |
| 1. Chae SH, Kim YL, Lee SM. Effects of phase proprioceptive training on balance in patients with chronic stroke. Journal of Physical Therapy Science. 2017; 29(5):839-44. | Intervention: manipulating sensory input |
| 1. Chan BKS, Ng SSM, Ng GYF. A home-based program of transcutaneous electrical nerve stimulation and task-related trunk training improves trunk control in patients with stroke: a randomized controlled clinical trial. Neurorehabilitation and Neural Repair. 2015; 29(1):70-9. | Intervention: stimulation elicits motor contraction |
| 1. Cho H, In TS, Cho KH, Song CH. A single trial of transcutaneous electrical nerve stimulation (TENS) improves spasticity and balance in patients with chronic stroke. Tohoku Journal of Experimental Medicine 2013; 229(3):187-93. | Intervention: stimulation elicits motor contraction |
| 1. Colomer C, Noe E, Llorens R. Mirror therapy in chronic stroke survivors with severely impaired upper limb function: a randomized controlled trial. European Journal of Physical and Rehabilitation Medicine. 2016; 52(3):271-8. | Intervention: mirror therapy |
| 1. Conforto AB, Ferreiro KN, Tomasi C, dos Santos RL, Moreira VL, Marie SKN, et al. Effects of somatosensory stimulation on motor function after subacute stroke. Neurorehabilitation and Neural Repair. 2010; 24(3):263-72. | Study design: not an RCT |
| 1. Conrad MO. Effects of distal sensory manipulations on arm movements in post-stroke hemiparesis: Marquette University; 2009. | No comparator |
| 1. DeSouza LH. The effects of sensation and motivation on regaining movement control following stroke. Physiotherapy. 1983; 69(7):238-40. | Study design: not an RCT |
| 1. Dewald JG, J. Head trauma and stroke. Reducing motor disability in hemiparetic stroke by manipulation of sensory input from the paretic upper limb: a quantitative evaluation. Rehabilitation R&D Progress Reports. 1997; 34:127-8. | Study design: not an RCT |
| 1. Dogru Huzmeli E, Yildirim SA, Kilinc M. Effect of sensory training of the posterior thigh on trunk control and upper extremity functions in stroke patients. Neurological Sciences. 2017; 38(4):651-7. | Study design: not an RCT |
| 1. Dohle C, Pullen J, Nakaten A, Kust J, Rietz C, Karbe H. Mirror therapy promotes recovery from severe hemiparesis: a randomized controlled. Neurorehabilitation and Neural Repair. 2009; 23(3):209-17. | Intervention: mirror therapy |
| 1. Eckhouse RHJ, Morash RP, Maulucci RA. Sensory feedback and the impaired motor system. Journal of Medical Systems. 1990; 14(3):93-105. | Study design: not an RCT |
| 1. Edmans JA, Webster J, Lincoln NB. A comparison of two approaches in the treatment of perceptual problems after stroke. Clinical Rehabilitation. 2000; 14:230-43. | Intervention |
| 1. Feys H, De Weerdt W, Verbeke G, Steck GC, Capiau C, Kiekens C, et al. Early and repetitive stimulation of the arm can substantially improve the long-term outcome after stroke: a 5-year follow-up study of a randomized trial. Stroke. 2004; 35:924-9. | Intervention: stimulation elicits motor contraction |
| 1. Floel A, Nagorsen U, Werhahn KJ, Ravindran S, Birbaumer N, Knecht S, et al. Influence of somatosensory input on motor function in patients with chronic stroke. Annals of Neurology. 2004; 56(2):206-12. | Study design: not an RCT |
| 1. Fong KN, Lo PC, Yu YS, Cheuk CK. Effects of sensory cueing on voluntary arm use for patients with chronic stroke: a preliminary study. Archives of Physical Medicine & Rehabilitation 2011; 92(1):15-23. | Study design: not an RCT |
| 1. Frolov AA, Mokienko O, Lyukmanov R, Biryukova E, Kotov S, Turbina L, et al. Post-stroke rehabilitation training with a motor-imagery-based brain-computer interface (BCI)-controlled hand exoskeleton: a randomized controlled multicenter trial. Frontiers in Neuroscience. 2017; 11(400):1-11. | Intervention: brain computer interface |
| 1. Fuller CF, Davies JM. The effect of vibration and transcutaneous electrical stimulation on postural control in a patient with stroke. Canadian Journal of Rehabilitation. 1988; 2(2):79-86. | Study design: not an RCT |
| 1. Goliwas M, Kocur P, Furmaniuk L, Majchrzycki M, Wiernicka M, Lewandowski J. Effects of sensorimotor foot training on the symmetry of weight distribution on the lower extremities of patients in the chronic phase after stroke. Journal of Physical Therapy Science. 2015; 27(9):2925-30. | Intervention: stimulation elicits motor contraction |
| 1. Gong W, Zhang T, Cui L, Yang Y, Sun X. Electro-acupuncture at Zusanli (ST 36) to improve lower extremity motor function in sensory disturbance patients with cerebral stroke: a randomized controlled study of 240 cases. Neural Regeneration Research. 2009; 4(11):935-40. | Intervention: acupuncture |
| 1. Gosman-Hedstrom G, Claesson L, Kilingenstierna U. Sensory stimulation for stroke rehabilitation: four randomised trials (A-D). Stroke. 1998; 29:2100-8. | Intervention: acupuncture |
| 1. Haral PP, Yardi S, Karajgi A. Effect of sensorimotor integration on Balance and Gait in chronic stroke patients. Indian Journal of Physiotherapy and Occupational Therapy. 2014; 8(1):64-9. | Intervention: manipulating sensory input |
| 1. Hayward KS, Barker RN, Brauer SG, Lloyd D, Horsley SA, Carson RG. SMART Arm with outcome-triggered electrical stimulation: a pilot randomized clinical trial. Topics in Stroke Rehabilitation 2013; 20(4):289-98. | Intervention: stimulation elicits motor contraction |
| 1. Huzmeli ED, Yildirim SA, Kilinc M. Effect of sensory training of the posterior thigh on trunk control and upper extremity functions in stroke patients. Neurological Sciences. 2017; 38(4):651-7. | Study design: not an RCT |
| 1. Hwang CH, Seong JW, Son D. Individual finger synchronized robot-assisted hand rehabilitation in subacute to chronic stroke: a prospective randomized clinical trial of efficacy. Clinical Rehabilitation. 2012; 26(8):696-704. | Intervention: robotic therapy |
| 1. Ibrahimi N, Tufel S, Singh H, Maurya M. Effect of sitting balance training under varied sensory input on balance and quality of life in stroke patients. Indian Journal of Physiotherapy and Occupational Therapy. 2010; 4(2):40-5. | Intervention: manipulating sensory input |
| 1. Jang SH, Lee J. Impact of sensory integration training on balance among stroke patients: sensory integration training on balance among stroke patients. Open Medicine. 2016; 11(1):330-5. | Intervention: FES |
| 1. Ji S, Cha H, Kim M, Lee C. The effect of mirror therapy integrating functional electrical stimulation on the gait of stroke patients. Journal of Physical Therapy Science. 2014; 26(4):497-9. | Intervention: FES |
| 1. Johansson BB, Haker E, von Arbin M, Britton M, Langstrom G, Terent A, et al. Acupuncture and transcutaneous nerve stimulation in stroke rehabilitation: a randomized, controlled trial. Stroke. 2001; 32(3):707-13. | Intervention: acupuncture |
| 1. Johansson K, Lindgren I, Widner H, Wiklund I, Johansson BB. Can sensory stimulation improve the functional outcome in stroke patients? Neurology. 1993; 43(11):2189-92. | Intervention: acupuncture |
| 1. Jung K, Kim Y, Cha Y, In T, Hur Y, Chung Y. Effects of gait training with a cane and an augmented pressure sensor for enhancement of weight bearing over the affected lower limb in patients with stroke: a randomized controlled pilot study. Clinical Rehabilitation. 2015; 29(2):135-42. | Intervention |
| 1. Kahn LE, Zygman ML, Rymer WZ, Reinkensmeyer DJ. Robot-assisted reaching exercise promotes arm movement recovery in chronic hemiparetic stroke: a randomized controlled pilot study. Journal of NeuroEngineering and Rehabilitation. 2006; 3(12):1-13. | Intervention: robotic therapy |
| 1. Kawahira K, Shimodozono M, Etoh S, Kamada K, Noma T, Tanaka N. Effects of intensive repetition of a new facilitation technique on motor functional recovery of the hemiplegic upper limb and hand. Brain Injury. 2010; 24(10):1202-13. | Study design: not an RCT |
| 1. Kim TH, In TS, Cho H. Task-related training combined with transcutaneous electrical nerve stimulation promotes upper limb functions in patients with chronic stroke. The Tohoku Journal of Experimental Medicine. 2013; 231(2):93-100. | Intervention: stimulation elicits motor contraction |
| 1. Kita K, Otaka Y, Takeda K, Sakata S, Ushiba J, Kondo K, et al. A pilot study of sensory feedback by transcutaneous electrical nerve stimulation to improve manipulation deficit caused by severe sensory loss after stroke. Journal of NeuroEngineering and Rehabilitation. 2013; 10(55):1-16. | Study design: not an RCT |
| 1. Krewer C, Hartl S, Müller F, Koenig E. Effects of repetitive peripheral magnetic stimulation on upper- limb spasticity and impairment in patients with spastic hemiparesis: a randomized, double-blind, sham-controlled study. Archives of Physical Medicine & Rehabilitation. 2014; 95:1039-47. | Intervention: stimulation elicits motor contraction |
| 1. Kumar V, Arya KN, Pandian S, Sethi T, Samal SK. Mirror therapy in management of somatosensory impairment among post stroke hemiparesis. International Journal of Stroke. 2016; 11:133. | Intervention: mirror therapy |
| 1. Kyoungsim J, Jinhwa J, Taesung I, Taehoon K, Hwi-young C. The influence of task-related training combined with transcutaneous electrical nerve stimulation on paretic upper limb muscle activation in patients with chronic stroke. NeuroRehabilitation. 2017; 40(3):315-23. | Intervention: stimulation elicits motor contraction |
| 1. Laddha D, Ganesh GS, Pattnaik M, Mohanty P, Mishra C. Effect of transcutaneous electrical nerve stimulation on plantar flexor muscle spasticity and walking speed in stroke patients. Physiotherapy Research International 2016; 21:247-56. | Intervention: stimulation elicits motor contraction |
| 1. Lämås K, Häger C, Lindgren L, Wester P, Brulin C. Does touch massage facilitate recovery after stroke? A study protocol of a randomized controlled trial. BMC Complementary and Alternative Medicine 2016; 16(50):1-9. | Progress Report |
| 1. Lima NM, Menegatti KC, Yu E, Sacomoto NY, Oberg TD, Honorato DC. Motor and sensory effects of ipsilesional upper extremity hypothermia and contralesional sensory training for chronic stroke patients. Topics in Stroke Rehabilitation. 2015; 22(1):44-55. | Study design: not an RCT |
| 1. Liu H, Au-Yeung SSY. Corticomotor Excitability Effects of Peripheral Nerve Electrical Stimulation to the Paretic Arm in Stroke. American Journal of Physical Medicine and Rehabilitation. 2017; 96(10):687-93. | Intervention: stimulation elicits motor contraction |
| 1. Liu S, Shi Z. Observation on the therapeutic effect of scalp acupuncture and body acupuncture in combination with rehabilitation exercise for hemiplegia and shoulder pain after stroke. World Journal of Acupuncture- Moxibustion. 2013; 23(1):21-6. | Intervention: acupuncture |
| 1. Magnusson M, Johansson K, Johansson BB. Sensory stimulation promotes normalization of postural control after stroke. Stroke. 1994; 25(6):1176-80. | Intervention: acupoint electrical stimulation |
| 1. Mastos M, Carey L. Occupation-based outcomes associated with sensory retraining post-stroke. International Journal of Stroke. 2010; 5(Suppl 1):29. | Study outcomes: aims |
| 1. McDonnell MN, Hillier SL, Miles TS, Thompson PD, Ridding MC. Influence of combined afferent stimulation and task-specific training following stroke: a pilot randomized controlled trial. Neurorehabilitation and Neural Repair. 2007; 21(5):435-43. | Intervention: stimulation elicits motor contraction |
| 1. McGuire J, Roth EJ. Head trauma and stroke. A controlled study of the effects of EMG feedback and electrical stimulation on motor recovery in acute stroke patients. Rehabilitation R&D Progress Reports. 1997; 34(126). | Progress Report |
| 1. Miller KJ, Galea P, Kilbreath SL, Phillips BA. Early intensive task-speciﬁc sensory and motor training of the upper limb after acute stroke: a pilot study. Neurorehabilitation and Neural Repair 2001; 15(4):345-6. | Intervention |
| 1. Ng SSM, Hui-Chan CWY. Transcutaneous electrical nerve stimulation combined with task-related training improves lower limb functions in subjects with chronic stroke. Stroke. 2007; 38(11):2953-9. | Intervention: acupuncture |
| 1. Ng SSM, Hui-Chan CWY. Does the use of TENS increase the effectiveness of exercise for improving walking after stroke? A randomized controlled clinical trial [with consumer summary]. Clinical Rehabilitation. 2009; 23(12):1093-103. | Intervention: acupoint electrical stimulation |
| 1. Paras J. A study to check added effects of electrical stimulation with task oriented training in hand rehabilitation among stroke patients. Indian Journal of Physiotherapy & Occupational Therapy. 2013; 7(1):154-9. | Intervention: stimulation elicits motor contraction |
| 1. Park M, Won J. The effects of task-oriented training with altered sensory input on balance in patients with chronic stroke. The Journal of Physical Therapy Science. 2017; 29:1208-11. | Intervention: manipulating sensory input |
| 1. Peurala SH, Pitkanen K, Sivenius J, Tarkka IM. Cutaneous electrical stimulation may enhance sensorimotor recovery in chronic stroke. Clinical Rehabilitation. 2002; 16(7):709-16. | Study design: not an RCT |
| 1. Platz T, Eickhof C, van Kaick S, Engel U, Pinkowski C, Kalok S, et al. Impairment-oriented training or Bobath therapy for severe arm paresis after stroke: A single-blind, multicentre randomized controlled trial. Clinical Rehabilitation. 2005; 19(7):714-24. | Intervention |
| 1. Pundik S, Scoco A, Skelly M, McCabe J, Daly JJ. Improvement of sensory function following rehabilitation in chronic stroke is associated with increased cortical thickness. American Heart Association/American Stroke Association 2015. | Study design: not an RCT |
| 1. Radajewska A, Opara JA, Kucio C, Błaszczyszyn M, Mehlich K, Szczygiel J. The effects of mirror therapy on arm and hand function in subacute stroke in patients. International Journal of Rehabilitation Research 2013; 36:268-74. | Intervention: mirror therapy |
| 1. Rowe JB, Chan V, Ingemanson ML, Cramer SC, Wolbrecht ET, Reinkensmeyer DJ. Robotic assistance for training finger movement using a Hebbian model: a randomized controlled trial. Neurorehabilitation and Neural Repair. 2017; 31(8):769-80. | Intervention: robotic therapy |
| 1. Sachdev HS. Development of auto repetitive interval electrical stimulation (ARIES) device for augmenting upper extremity function after stroke. International Journal of Stroke. 2016; 11(Suppl 3):135-6. | Intervention: robotic therapy |
| 1. Samuelkamaleshkumar S, Reethajanetsureka S, Pauljebaraj P, Benshamir B, Padankatti SM, David JA. Mirror therapy enhances motor performance in the paretic upper limb after stroke: a pilot randomized controlled trial. Archives of Physical Medicine & Rehabilitation. 2014; 95(11):2000-5. | Intervention: mirror therapy |
| 1. Sargolzaei K, Fallah MS, Aghebati N, Esmaily H, Farzadfard MT. Effect of a structured sensory stimulation program on the sensory function of patients with stroke-induced disorder of consciousness. Evidence Based Care 2017; 7(2):7-16. | Intervention |
| 1. Sehle A, Busching I, Dinse HR, Liepert J. Passive repetitive sensory electrical stimulation to improve motor function after stroke - a pilot study. Neurologie und Rehabilitation. 2017; 23(2):179-83. | Study design: not an RCT |
| 1. Semrau JA, Herter TM, Scott SH, Dukelow SP. Examining differences in patterns of sensory and motor recovery after stroke with robotics. Stroke. 2015; 46(12):3459-69. | Study design: not an RCT |
| 1. Sheffler LR, Taylor PN, Bailey SN, Gunzler DD, Buurke JH, Ijzerman MJ, et al. Surface peroneal nerve stimulation in lower limb hemiparesis: effect on quantitative gait parameters. American Journal of Physical Medicine & Rehabilitation 2015; 94(5):341-57. | Intervention: FES |
| 1. Sonde L, Gip C, Fernaeus SE, Nilsson CG, Viitanen M. Stimulation with low frequency (1.7 Hz) transcutaneous electric nerve stimulation (low-tens) increases motor function of the post-stroke paretic arm. Scandinavian Journal of Rehabilitation Medicine. 1998; 30(2):95-9. | Intervention: stimulation elicits motor contraction |
| 1. Sonde L, Kalimo H, Fernaeus S, Viitanen M. Low TENS treatment on post-stroke paretic arm: a three-year follow-up. Clinical Rehabilitation. 2000; 14(1):14-9. | Study outcomes: aims |
| 1. Song YB, Chun MH, Kim W, Lee SJ, Yi JH, Park DH. The effect of virtual reality and tetra-ataxiometric posturography programs on stroke patients with impaired standing balance. Annals of Rehabilitation Medicine. 2014; 38(2):160-6. | Intervention |
| 1. Sullivan J, Lopez-Rosado R. Sensory amplitude electrical stimulation improves gait speed in chronic stroke...2016 ACRM / American Congress of Rehabilitation Medicine Annual Conference Archives of Physical Medicine & Rehabilitation 2016; 97(10):27. | Study design: not an RCT |
| 1. Suzuki K. Effect of repetitive peripheral magnetic stimulation for lower extremity of stroke patients with hemiplegia. Japanese Journal of Comprehensive Rehabilitation Science. 2015; 6:56-63. | Intervention: stimulation elicits motor contraction |
| 1. Swanton R, Doyle S, Bennett S. Four weeks (10 sessions) of individual sensory discrimination training produced clinically important changes in upper limb sensation after stroke. Australian Occupational Therapy Journal. 2012; 59(2):165-7. | Summary of an included RCT |
| 1. Tanaka T, Takeda H, Yumoto H, Ino S, Ifukube T. Improvement on toe tactile sensation of the great toe for re-establishing standing balance in hemiplegic subjects. Journal of Human Movement Studies. 1998; 35(4):151-65. | Study design: not an RCT |
| 1. Thielbar KO, J. LT, Fischer HC, Lazzaro EC, Barth KC, Stoykov ME, et al. Training finger individuation with a mechatronic-virtual reality system leads to improved fine motor control post-stroke . Journal of NeuroEngineering and Rehabilitation. 2014; 11(171):1-11. | Intervention: visual stimulation |
| 1. Tyson S, Wilkinson JA, Thomas N, Selles RW, McCabe C, Tyrrell P, et al. Phase II pragmatic randomized controlled trial of patient-led therapies (mirror therapy and lower-limb exercises) during inpatient stroke rehabilitation. Neurorehabilitation and Neural Repair. 2015; 29(9):818-26. | Intervention: mirror therapy |
| 1. Valentini M, Kischka U, Halligan PW. Residual haptic sensation following stroke using ipsilateral stimulation. Journal of Neurology, Neurosurgery, and Psychiatry. 2008; 79(3):266-70. | Not an intervention |
| 1. Van Nes IJW, Latour H, Schils F, Meijer R, Van Kuijk A, Geurts ACH. Long-term effects of 6-week whole-body vibration on balance recovery and activities of daily living in the post-acute phase of stroke: A randomized, controlled trial. Stroke. 2006; 37(9):2331-5. | Intervention |
| 1. Vinograd A, Taylor D, Grossman S. Sensory retraining of the hemiplegic hand. American Journal of Occupational Therapy 1962; 16(6):246-50. | Study design: not an RCT |
| 1. Volpe B, Krebs H, Edelstein L, McDowell F, Hogan N. Robot aided sensorimotor stimulation as a novel approach to stroke rehabilitation: Experience to date. Stroke. 2000; 31(2768-2871). | Intervention: robotic therapy |
| 1. Wilson RD, Page SJ, Delahanty M, Knutson JS, Gunzler DD, Sheffler LR, et al. Upper-limb recovery after stroke: a randomized controlled trial comparing EMG-triggered, cyclic, and sensory electrical stimulation. Neurorehabilitation and Neural Repair. 2016; 30(10):978-87. | Intervention: NMES |
| 1. Winward CE, Haligan PW, Wade DT. Somatosensory recovery: longitudinal study of the first 6 months after unilateral stroke. Disability & Rehabilitation. 2007; 29(4):293-9. | Study design: not an RCT |
| 1. Wolny T, Saulicz E, Gnat R, Kokosz M. Butler's neuromobilizations combined with proprioceptive neuromuscular facilitation are effective in reducing of upper limb sensory in late-stage stroke subjects: a three-group randomized trial. Clinical Rehabilitation. 2010; 24(9):810-21. | Intervention |
| 1. Yamaguchi T, Tanabe S, Muraoka Y, Masakado Y, Kimura A, Tsuji T, et al. Immediate effects of electrical stimulation combined with passive locomotion-like movement on gait velocity and spasticity in persons with hemiparetic stroke: a randomized controlled study [with consumer summary]. Clinical Rehabilitation. 2012; 26(7):619-28. | Intervention: FES |
| 1. Yang H, Liu T, Wang Y, Ying S, Zheng C, Kuai L, et al. Acupoint electrogymnastics therapy in stroke hemiplegia. Neural Regeneration Research 2008; 3(10):1145-51. | Intervention: acupoint electrical stimulation |
| 1. Yasuda K, Kaibuki N, Harashima H, Iwata H. The effect of a haptic biofeedback system on postural control in patients with stroke: An experimental pilot study. Somatosensory & Motor Research. 2017; 34(2):65-71. | Study design: not an RCT |
| 1. Yasuda K, Kaibuki N, Watanabe S, Harashima H, Iwata H. A vibro-tactile biofeedback system supplying online center of foot pressure displacement for balance training in stroke patients. WCPT Congress/Physiotherapy. 2015; 101(Suppl 1):1687-8. | Study design: not an RCT |
| 1. Yekutiel M, Guttman E. A controlled trial of the retraining of the sensory function of the hand in stroke patients. Journal of Neurology, Neurosurgery & Psychiatry 1993; 56(3):241-4. | Study design: not an RCT |
| 1. Yozbatiran N, Donmez B, Kayak N, Bozan O. Electrical stimulation of wrist and fingers for sensory and functional recovery in acute hemiplegia. Clinical Rehabilitation. 2006; 20(1):4-11. | Study design: not an RCT |
